# Mammalian Collection on Noah's Ark: The Effects of Beauty, Brain and Body Size

**DOI:** 10.1371/journal.pone.0063110

**Published:** 2013-05-15

**Authors:** Daniel Frynta, Olga Šimková, Silvie Lišková, Eva Landová

**Affiliations:** Department of Zoology, Faculty of Sciences, Charles University in Prague, Prague, Czech Republic; University of Kent, United Kingdom

## Abstract

The importance of today's zoological gardens as the so-called “Noah's Ark” grows as the natural habitat of many species quickly diminishes. Their potential to shelter a large amount of individuals from many species gives us the opportunity to reintroduce a species that disappeared in nature. However, the selection of animals to be kept in zoos worldwide is highly selective and depends on human decisions driven by both ecological criteria such as population size or vulnerability and audience-driven criteria such as aesthetic preferences. Thus we focused our study on the most commonly kept and bred animal class, the mammals, and we asked which factors affect various aspects of the mammalian collection of zoos. We analyzed the presence/absence, population size, and frequency per species of each of the 123 mammalian families kept in the worldwide zoo collection. Our aim was to explain these data using the human-perceived attractiveness of mammalian families, their body weight, relative brain size and species richness of the family. In agreement with various previous studies, we found that the body size and the attractiveness of mammals significantly affect all studied components of the mammalian collection of zoos. There is a higher probability of the large and attractive families to be kept. Once kept, these animals are presented in larger numbers in more zoos. On the contrary, the relative mean brain size only affects the primary selection whether to keep the family or not. It does not affect the zoo population size or the number of zoos that keep the family.

## Introduction

Nowadays, mankind covers about 83% of the Earth's land surface [1], causing global biodiversity to decline due to a quick loss of natural habitats of many species [2]. The proportion of potentially threatened species is rapidly increasing, leaving only very few species safe from a possible extinction [3]. It is therefore important not to miss any potential chance for animal conservation, including both in-situ and ex-situ conservational efforts. The world's zoos, aquaria, botanical gardens, and gene banks provide insurance for species and genetic diversity [4]. According to the ISIS (International Species Information System) online database, more than 7 million individual animals are kept in 872 zoos and aquariums (as recorded by the date of 12^th^ January 2011). The high potential of zoos to serve as wildlife reservoirs, coupled with the rapid destruction of nature that we have faced in the last few decades, led [5] to frame the landmark Ark Hypothesis. The role of zoos as an ark proved viable in the case of amphibians (the “Amphibian Ark”) that suffered a rapid population decline due to the chytridiomycosis disease. In response to this threat, The World Association of Zoos and Aquariums (WAZA), the IUCN/SSC (The International Union for Conservation of Nature/Species Survival Commission) Amphibian Specialist Group, and the IUCN/SSC Conservation Breeding Specialist Group (CBSG) worked together to collect a large number of species [6]. Moreover, in 1993 the EU recognized this conservation potential of zoos during the Convention on Biological Diversity (CBD), and obliged the zoos to manage the ex-situ and in-situ conservational role under the CBD's requirements [7], regardless of the lack of the government's systematic financial or other support [8]. EAZA (American Association of Zoos and Aquariums) and AZA (American Association of Zoos and Aquariums) supervise many specific ex-situ conservation programs such as Species Survival Plans and European Endangered Species Programmes. Moreover, they cooperate with CBSG, TAGs (Taxon Advisory Groups) and use various studbooks and data management systems, notably ISIS, to maintain the breeding of a variety of species which may also raise the effectiveness and possibility of species survival through captive breeding [9]–[11].

However, although the space to accommodate wild animals in zoos worldwide altogether is large, it is still very limited when compared to the list of all extant species. Only a small fraction of the world's animal population can board the Ark. In the year 2009, the Ark provided space for about 152 thousand individual mammals belonging to 990 species (18.5% of extant species) within a median worldwide zoo population size of 34.5 individuals. Empirical studies suggest that the minimum population size necessary for short-time captive maintenance of animal species/breeds under controlled conditions is about 50 [12], and populations over about 500 individuals are usually not affected by inbreeding depression [13]. Although these thresholds are only rough estimates because the effect depends on both effective population size and frequency of deleterious recessive mutations (cf. [14]–[16]). The zoos worldwide maintain 416 mammalian species represented by more than 50 individuals, which is 7.8% of all extant species. For the threshold of 500 individuals, the numbers count only 79 mammalian species, representing 1.5% of the extant mammalian diversity [17]. Similar numbers were confirmed in an independent study from April 2010 [18], finding that out of all 142 threatened mammalian species belonging to the IUCN categories Endangered, Critically Endangered, and Extinct in the wild, 68 species are being kept in zoos in more than 50 individuals, out of which 30 species are kept in more than 250 individuals.

Such small numbers point out that, even theoretically, if zoos tried to keep and breed endangered species, the space would be limited to hold only a tiny fraction of needful species at populations large enough to sustain a long-term captive breeding program of animals while avoiding an inbreeding depression. However, the presence of just a few unrelated individuals in zoos may occasionally save the species if the captive population is immediately expanded when necessary, e.g., after an unexpected crisis of the wild populations (but see [19] for negative effects of bottlenecking). This suggests that cooperative ex-situ conservation can help restore animal populations once the threat has diminished. Although many authors have questioned this assumption ever since (i.e., [20]–[24]), many researchers find the theoretical role of zoos in reintroduction programs as feasible (for a review of the limitations and solutions of availability of captive populations in reintroduction, see [25]; e.g., disease risk, behavioral competence of captive-reared individuals such as reduced ability to avoid predators or find food resources and attachment to humans; changes in genetic compositions, etc.), and a notable contribution of zoos to animals ex-situ breeding can be demonstrated by successful reintroductions which reduced the threat level of particular species. Species such as the Przewalski horse (*Equus ferus przewalskii*; [26]), the American bison (*Bison bison*; [27]), the European wisent (*Bison bonasus*; [28]–[30]), Pére David's deer (*Elaphurus davidianus*; [31]), or Arabian oryx [32] may serve as examples of successfully maintained zoo animal populations released back into the wild. Other notably successful reintroduced animals were the Asiatic wild ass (*Equus hemionus*; [33]), the golden lion tamarin (*Leontopithecus rosalia*; [34]), and the black-footed ferret (*Mustela nigripes*). The latter recovered from a very small population of only eighteen remaining individuals ([35]).

Moreover to the ex-situ breeding role of captive zoo populations, these groups might contribute to conservation purposes in other ways that do not necessarily demand high captive population densities. A very important role of today's zoological gardens presents education, especially promotion of increased public and political awareness of the need for in-situ conservation [36].

A single popular animal (or a small group of these) might serve as a flagship species and help its endangered relatives in the wild, or their natural habitat and its residents, to gain the necessary financial support from the public [37]. Another significant role of zoos resides in training specialists with the right knowledge about the breeding and care of the animals [38]. International studbooks running under the World Association of Zoos and Aquariums (WAZA) and collaboration with the ISIS database include husbandry and veterinary guidance for as many species as possible (www.waza.org). However, keeping a few individuals of a rare species, or their relatives, may help to retain the right specialists for future needs. The experience of staff and researchers working with living animals is irreplaceable by studbook information and guidelines, and their presence may help to save the species. Similarly, imagine a well-educated surgeon with no experience with real patients to perform operations on living humans.

The situation is complicated due to the fact that conservation activities are not the only purpose of the zoos. In fact, it is an issue discussed more intensively during the last several decades [38]. Zoos are vitally dependant on the funds gained from visitors [39]. Guests come to zoos mainly for recreational activities, expecting to see large, attractive, and active animals [40]–[42]. This may seemingly lead to the conclusion that there is a trade-off of which animals to keep in zoos to satisfy both conservational purposes and the visitors' recreational desires. The species' conservational status according to the IUCN was not the key factor for the selection of species to be included in the worldwide zoo collection (see [17], [18] for terrestrial vertebrates; [43] for parrots, [44] for boid snakes). A question thus arises: is it the attractiveness and/or factors connected with the attractiveness of the animals that determines the composition of the collection of zoo animals around the world?

Balmford et al. [45] hypothesized that it is the size of the animal that determines its presence in a zoo and we confirmed this relationship in almost all examined taxa of terrestrial vertebrates in our previous papers [17], [43], [44]. Additionally, we found that attractiveness of the animals to human respondents also affects the world's zoo population numbers in some clades, namely snakes [44], parrots [43], terrestrial birds (grouping followed [46], excluding Accipitriformes, Strigiformes, Trogonidae and Coliidae; Psittaciformes, Opisthocomus and Cuculiformes were added), basal mammals (Monotremata, Marsupialia, Xenarthra and Afrotheria), and mammals of the carnivore-ungulate clade (Laurasiatheria, [17]).

On principle, larger animals are more conspicuous and visible to potential zoo visitors. This leads to the assumption that size can be difficult to separate from attractiveness, yet still there is more to beauty than size itself. In this paper, we measured separate data of attractiveness perceived by human respondents for a nearly complete set of mammalian families. We hypothesized that both size and attractiveness of mammals are good predictors of the size of their captive zoo populations (World Zoo Collection; further referred to as WZC).

Moreover to the above-mentioned physical properties, the brain size may modulate the attractiveness of animals to zoo curators and visitors. Adolf Portmann, the well-known Swiss biologist of the 20^th^ century, hypothesized that humans categorize animals into the “higher” and “lower” ranked animal groups [47], and that this categorization, reflecting the brain size of the animal, affects the perceived attractiveness. Accordingly, we hypothesized that the brain size is a good predictor of behavioral attractiveness of mammalian species.

In this paper, we aimed to analyze the effect of body size, brain size, and attractiveness of mammalian species from almost all recent families to several variables explaining the WZC. We elaborate the previously-questioned number of individuals in the WZC in separate analyses to ask which factors determine whether the animal is present in any zoo or not, and to ask how many zoos actually keep those selected ones, and in how large or small numbers. There is a possibility that rather than species recognized by current taxonomists, taxa closer to genera or families represent the primary units of human spontaneous categorization [48]. Thus, the species within a family may compete with each other for the space available on the Ark (e.g., a zoo might select just one “mouse” or “rat” to keep in its collection instead of all 715 species of the family Muridae). Because of that, we included the species richness of the family as another factor to explain the analyzed WZC variables.

## Materials and Methods

The dataset used for statistical analyses (see Appendix S1) includes 123 rows referred to as families. There were 119 families recognized by [49] and four infraorders (i.e., Microchiroptera, Megachiroptera, Mysticeti, Odontoceti). Cetaceans and chiropterans were pooled into infraorders because these specialized aerial and marine taxa deviate considerably from the typical mammalian body plan and their representation in zoos is rather poor.

### Dependent variables

Information about the numbers of mammalian species and individuals kept in zoos worldwide was obtained from the ISIS (International Species Information System) online database (http://www.isis.org) covering more than 800 zoos and aquariums from 76 countries. We excluded hybrids, ambiguous genera, and domestic species/forms (see Appendix S2) from the dataset. We analyzed the following four variables derived from this dataset (accessed on 12^th^ January 2011):

1. **Proportion of zoo species.** The number of species kept in the WZC scaled to the total number of extant species of a given family. This binomial variable reflects the mean probability that a species is kept at least in one zoo.
2. **Mean population size.** The world zoo population size (square-root transformed) per species present in the WZC (the families not represented in the WZC were excluded).
3. **Number of zoos.** The natural log-transformed mean number of zoos keeping the species (species and families not represented in the WZC were excluded).
4. **Number of conspecifics per zoo.** The mean number of conspecifics per zoo keeping the species (the families not represented in the WZC were excluded).

### Explanatory variables

#### Species richness

The number of extant species of each family was log-transformed. In the case of infraorders, the number of extant species was divided by the number of families belonging to the infraorder.

#### Body size

We gathered body weight records (in grams) for representatives of most mammalian genera from literary sources (mostly from [50]). These values were naturally log-transformed and used for computation of family means.

#### Brain size

**Figure 1:**
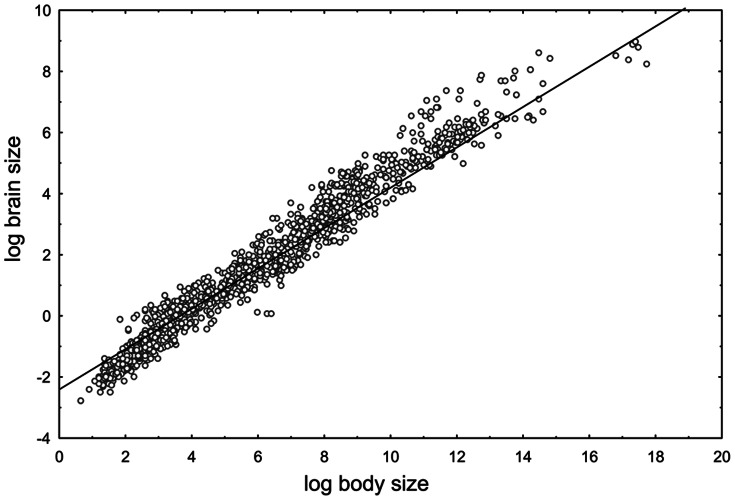
The allometric relationship between the brain and body sizes in 1309 mammalian species. Because the families were represented by an unequal number of data points (species), each family was given an equal weight when calculating the ordinary least-square regression. This correction for biased representation of the families resulted in a line seemingly unfitting the data points (representing species and not families). This adjusted regression line is considerably more reliable for further GLM analyses performed between family level, though. Allometric equation: ln(brain mass) = −0.6601 * ln(body mass)−2.4100.

The relative brain size was substituted by an encephalization quotient. This variable, introduced by [51]–[53] and then repeatedly used as a measure of relative brain size (e.g. [54]), is a natural log-transformed ratio between observed brain weight and theoretical brain weight predicted by an allometric equation for mammalian species of the given body size. We performed an ordinary least square regression to calculate the allometric relationship between brain and body size. We gathered the primary data on brain and body weights for 1309 mammalian species from various literary sources (see Appendix S3). Because the individual families were unequally represented in the dataset, we employed a weighting by the variable inversely proportional to the number of included species belonging to the particular family (Figure 1). The resulting empirical allometric equation (lnB = −0.6601*lnM-2.4100; B = brain mass, M = body mass) was used for a calculation of the encephalization quotients.

#### Attractiveness

**Figure 2:**
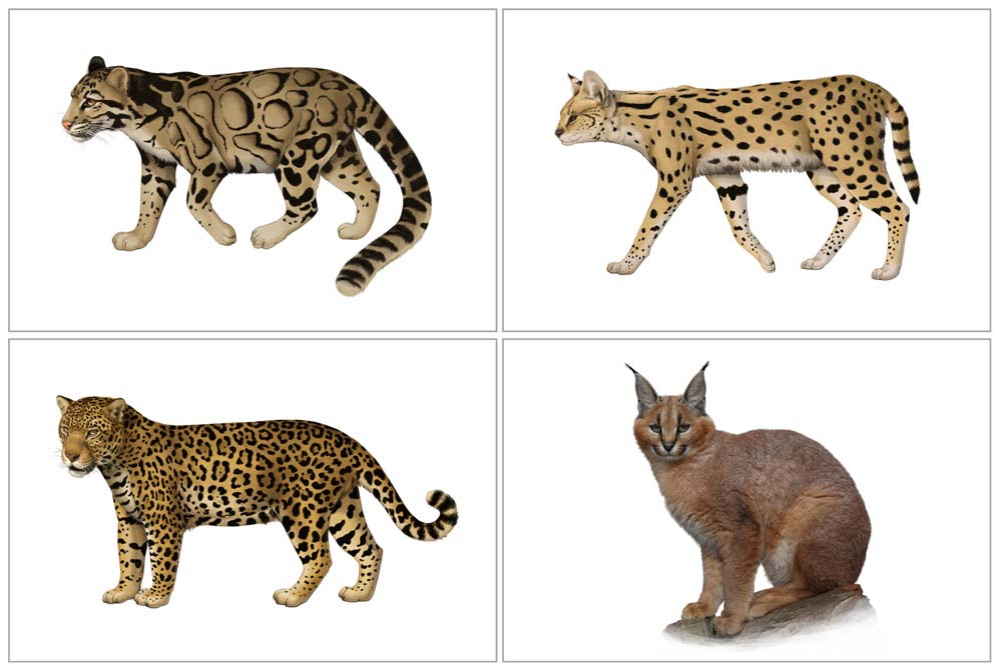
The pictures of the representatives of the family Felidae (the second most preferred family out of 123 examined ones). It illustrates the variation of body positions among four tested sets of pictures (for details see under Materials and Methods): clouded leopard *Neofelis nebulosa* (top left), serval *Leptailurus serval* (top right), jaguar *Panthera onca* (bottom left; pictures painted by Silvie Lišková) and caracal *Caracal caracal* (bottom right; photo from the archive of the Zoo Prague).

For the purpose of data collection, we defined four sets of 123 pictures depicting species from each family. Species representing individual families in each partial set were selected by a two-step (first genus then species) random choice process from the list of extant genera and species ([49]; domestic forms were excluded). Thus, duplicated presence of identical genera and species was avoided whenever possible. In monotypic families, the species was represented by different pictures. Sets coded as A, B and C consisted of illustrations while D consisted of photographs. The main sources of the pictures were [55]–[58]. In order to avoid possible effects of body size and background on rating, we adjusted the pictures with a white background and we resized them so that the pictured mammals were of a similar relative size (for illustration, see Figure 2).

The aesthetic attractiveness of the families was examined by presenting pictures of mammalian species to human volunteers (following [44]). The respondents were Czech citizens, mostly university students within the age range of 19–29 years. One can argue that as far as age, sex, and ethnic composition is concerned, our respondents did not properly represent the full scope of zoo visitors. The Czech Republic belongs among the least socially stratified countries in the world as demonstrated by the Gini index (GI) measuring the extent to which the distribution of income among individuals within an economy deviates from a perfectly equal distribution (0 corresponds to perfect equality while 100 to perfect inequality). The GI of the Czech Republic is 31 which corresponds to the 110 rank of 137 countries included in a comparison provided by the World Bank ([59]; compare, e.g., with GI = 45 for the USA which places them to the 41st position in the ranking; the higher the rank, the higher is the equality in society). Thus, we expected the Czech students to possess aesthetic preferences for animals comparable to the rest of the society, including zoo visitors (but see [60], [61] who found socio-economic and educational differences in preferences, but their method did not focus purely on aesthetics). Since we preferred a homogenous example of respondents well motivated to performing the task, in which we could focus on the variables related to the tested stimuli rather than respondent characteristics, the students presented a good testing sample for the experiments evaluating human-perceived animal attractiveness.

Moreover, our previous studies revealed that the aesthetic ranking of animal species is highly stable with the factors of age, sex and ethnic composition having only a marginal effect ([43]). This is especially surprising in the case of cross-cultural comparisons, e.g., in such different cultures as are those in Europe and Papua New Guinea ([62], [63] and new unpublished data; see also the agreement in physical attractiveness ratings of female faces across cultures [64], but see [65]). Note that the sample of respondents from non-European countries differed greatly in age and/or socioeconomic rank but their preferences for the examined animals still highly corresponded to those of the Czech students.

During the experiment, each respondent was exposed to one set of 123 pictures that were placed on a table in a random assemblage. Their objective was to “pack the photographs in an order corresponding to the beauty of the depicted species from the most beautiful to the least beautiful one”, as we asked them. The order of the pictures in the pack was then coded by numerals from 1 (the most beautiful one) to 123, further referred to as ranks. Although no explicit time limit was given, all the respondents performed the task in about 30 minutes. Each set of pictures was evaluated by a comparable number of respondents: 77 (25 men), 85 (31), 77 (27) and 75 (25) for sets A, B, C and D, respectively. Altogether, we gathered data from 314 respondents; 206 of which were women and 108 were men.

All respondents agreed to participate in the project voluntarily. Each subject provided a written informed consent and additional information about gender, age and their affinity to mammals. The age and gender had no effect on the preferences (MANOVA, all Ps>0.05), which allowed us to pool the data. The affinity to mammals exhibited too low of a variance (91.85% respondents reported positive affinity to mammals, 8.15% neutral and 0 negative) to allow reliable testing. The experiments were performed in accordance with the European law and were approved by The Institutional Review Board of Charles University, Faculty of Science (No. 2009/2).

In order to quantify and test congruence in species ranking provided by different respondents and/or to compare sets composed of different species, we adopted Kendall's Coefficient of Concordance (W) as implemented in SPSS v.16.0 [66]. There was considerable congruence among the respondents in all four sets of pictures; W coefficients were 0.206, 0.264, 0.224 and 0.334 for sets A, B, C and D, respectively (all p<0.001).

Prior to further analyses, the raw ranks were transformed as follows: each value was divided by the number of evaluated families (123) and square-root arcsine transformed to improve its statistical distribution. Next, we computed the mean transformed rank for each set and family. Mean transformed ranks computed for individual sets were mutually significantly correlated (r^2^ = 0.334, 0.450, 0.401, 0.324, 0.449 and 0.420 for A vs. B, A vs. C, A vs. D, B vs. C, B vs. D and C vs. D, respectively; all p<0.0001). This allowed us to compute family means from the mean preference ranks obtained for partial sets of pictures and to further use this variable as a simplified measure of aesthetic attractiveness of the family for humans. The numeric values of this variable were positivized (multiplied by −1) to make the explanation of the results more intuitive.

We included another explanatory variable in preliminary analyses: whether the family is mainly diurnal or nocturnal/fossorial. However, this variable correlates with the body size (r = −0.51), and when the influence of body size is removed, the factor itself explains neither of the analyzed dependant variables. As such, we removed it from further analyses.

### Statistical treatment

In order to examine the effects of species richness, body size, attractiveness, and brain size on dependent variables, we generated General Linear Models (GLMs) in R 2.8.0 [67]. In the case of the proportion of zoo species, we adopted the binomial model with logit link function and Chi – square tests. We used the Gaussian distribution with identity link in remaining analyses. AIC criterion was used to reduce the original full models. The simplified model was also compared to the previous model by the ANOVA test to verify that the change in residual deviance was not significant (P>0.05).

Because species data are not independent as a result of shared phylogeny among more closely related taxa [68], we also carried out a phylogenetically controlled analysis using the independent contrast method [69]. For the purpose of this analysis, we used a phylogenetic tree of families compiled from recent studies dealing with molecular phylogenies. The main branching was adopted from Bininda-Emonds [70] and Arnason et al. [71], while specialized studies were used to improve the branching of partial crown taxa: Marsupialia [72], Xenarthra [73], Cetartiodactyla [74], Carnivora [75], Madagascar carnivores [76], Rodentia [77], Hystricognathi [78], Platacanthomyidae [79], Muridae [80], and Primates [81]. When phylogenetic information was equivocal, we resolved the tree in accordance with the conventional taxonomy. The independent contrasts of the arcsine-transformed mean preference rank, log-transformed WZPS, species number and body mass were computed using COMPARE, version 4.6b [82]. All branch lengths were set to 1 because the corresponding estimations were not available. Thus, for the contrasts analysis, we assumed the applicability of the punctuational model of evolution. The diagnostic proposed by [83] revealed that the contrasts were appropriately standardized. The multiple regression analyses based on independent contrast scores were performed in Statistica 6.0. [84] and constrained to pass through the origin [83].

## Results

The analyzed zoos kept 179 868 mammals belonging to 1048 species and 103 families. The most represented families belonged predominantly to the carnivore-ungulates, primates and Xenarthra-Afrotheria clades (Appendix S1).

The highest aesthetic attractiveness was found in the large sized mammals (families Ailuridae, Felidae, Phascolarctidae, Ursidae, Giraffidae, Elephantidae, Equidae, Macropodidae, Mephitidae, and Cervidae). In contrast, the least preferred mammals (families Notoryctidae, Bathyergidae, Chrysochloridae, Spalacidae, Caenolestidae, Solenodontidae, Talpidae, Ctenomyidae, Geomyidae, and Dasypodidae) were predominantly small subterranean (fossorial) creatures with reduced eyes.

### Proportion of zoo species

**Figure 3:**
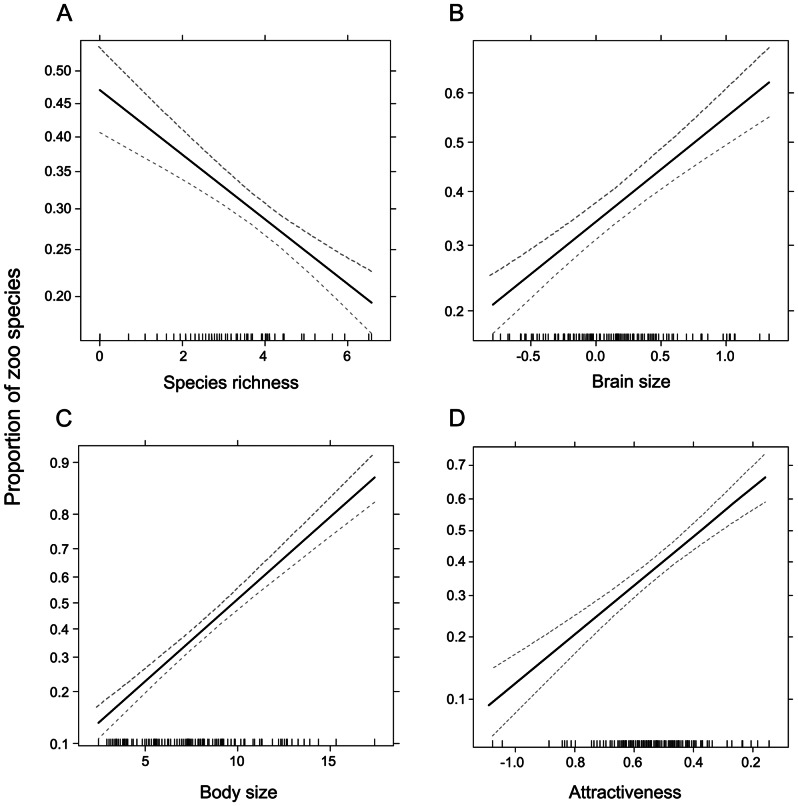
The proportion of zoo species as predicted by GLM. The effects of species richness (a), brain size (b), body size (c), and attractiveness (d). The dependent variable is the number of species kept in WZC scaled to the total number of extant species of a given family. This binomial variable reflects the mean probability that a species is kept in at least one zoo. For the definition and transformation of the explanatory variables see under the Material and methods section.

GLM revealed that the proportion of zoo species is associated positively with body size, attractiveness, and brain size, and negatively with species richness (all Ps<0.0001). This result was confirmed when the original variables were replaced by their independent contrasts and treated by a multiple regression through the origin (Table 1a, Figure 3).

**Table 1:** The parameters of the reduced general linear models examining the effects of species richness, body size, brain size and attractiveness on dependent variables reflecting the representation of mammalian species in WZC.

A.	Anova					Coefficients	Independent contrasts		
	Df	Deviance	Resid.Df	Resid.Dev	P	Estimate	Beta	B	p-level
NULL	122	1855.22							
(Intercept)						−0.447			
Taxonomic uniqueness	1	333.04	121	1522.18	<0.0000	−0.199	−0.309	−0.063	0.0001
Body size	1	770.76	120	751.42	<0.0000	0.257	0.363	0.059	<0.0000
Brain size	1	81.73	119	669.69	<0.0000	0.861	0.176	0.165	0.0221
Attractiveness	1	71.33	118	598.36	<0.0000	3.178	0.242	0.582	0.0020

B.	Anova					Coefficients	Independent contrasts		
	Df	Sum Sq	Mean Sq	F value	P	Estimate	Beta	B	p-level
(Intercept)						11.152			
Body size	1	383.31	383.31	16.9380	0.0001	0.468			
Attractiveness	1	272.03	272.03	12.0210	0.0008	12.499	0.389	16.375	<0.0000
Residuals	100	2263	22.63						

C.	Anova					Coefficients	Independent contrasts		
	Df	Sum Sq	Mean Sq	F value	P	Estimate	Beta	B	p-level
(Intercept)						2.336			
Body size	1	44.23	44.23	38.2475	<0.0000	0.188	0.237	0.145	0.0104
Attractiveness	1	9.75	9.75	8.4308	0.0045	2.366	0.350	3.416	0.0002
Residuals	100	115.65	1.16						

D.	Anova					Coefficients	Independent contrasts		
	Df	Sum Sq	Mean Sq	F value	P	Estimate	Beta	B	p-level
(Intercept)						3.663			
Taxonomic uniqueness							−0.209	−0.152	0.0318
Body size	1	11.02	11.02	10.5723	0.0016	−0.129	−0.370	−0.193	0.0002
Attractiveness	1	2.17	2.17	2.0789	0.1525	−0.001			
Residuals	100	104.20	1.04						

a) Proportion of extant species kept in WZC.

b) World zoo population size per species in WZC.

c) Proportion of zoos keeping the species.

d) Individuals per Zoo Keeping the species.

The results of the phylogenetically adjusted analyses (multiple regression of independent contrasts performed through the origin) are also included. For the definition and transformation of the variables see under the Material and methods section.

### Mean population size

**Figure 4:**
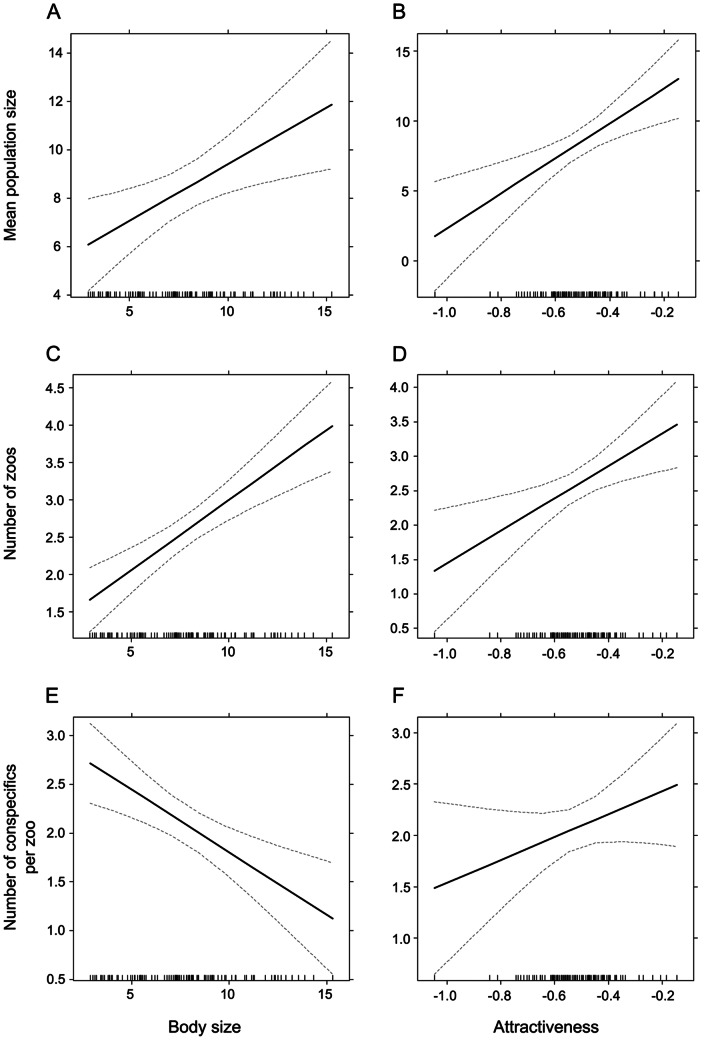
The effects of body size and attractiveness. The effects of body size and attractiveness on the mean population size (a,b), number of zoos (c,d) and number of conspecifics per zoo (e,f). The mean population size: The world zoo population size (square-root transformed) per species present in WZC. The number of zoos: The natural log-transformed mean number of zoos keeping the species. The number of conspecifics per zoo: The mean number of conspecifics per zoo keeping the species. The families not represented in WZC were excluded from the analyses. For the definition and transformation of the explanatory variables see under the Material and methods section.

Mean population size of zoo species was associated positively with body size (P = 0.0001) and attractiveness (P = 0.0008). Only the effect of attractiveness was confirmed by the independent contrast analysis (P<0.0001; Table 1b, Figure 4a,b).

### Number of zoos

Both GLM of original data and multiple regression of independent contrasts agreed that the mean proportion of zoos keeping the species was significantly predicted by the body size (P<0.0001 and P = 0.0104, respectively) and attractiveness (P = 0.0045 and P = 0.0002, respectively) of the animal (Table 1c, Figure 4c,d).

### Number of conspecifics per zoo

GLM revealed negative effect of body size (P = 0.0016) on the mean number of conspecifics kept in a zoo. This effect was confirmed by multiple regression of independent contrasts (P = 0.0002). The latter analysis also revealed negative effect of species richness (P = 0.0318; Table 1d, Figure 4e,f).

## Discussion

We analyzed the representation of mammalian species in the WZC in the year 2011 and we found that it was very poor and highly selective, comparable to its state in 2009. [18] The list of currently recognized mammalian species [49] contains 5334 extant species, but only 1048 of them (16%) were actually present in the WZC. There were twenty families that were entirely absent in the WZC. Such selectivity may not only affect the putative conservation value of zoo populations, but it may also warn us about the existence of a large bias in species selection for conservation in general.

The selection of species into the WZC is determined by decisions made by humans, and although the selection criteria might be different, we may still assume that the psychological drives behind such selection are the same or similar to those for the selection of species for conservation and reintroduction. Such conclusion is supported by the fact that, between the years 1992–2009, out of the 12 evaluated reintroduced mammalian families, 11 of them are in the top-half when taking brain size (EQ) or attractiveness into account (for the list of reintroduced families with detailed published results, see Appendix S4). The “intelligent” and “beautiful” animals seem to be favored in human decision-makings. Moreover, many conservation programs strongly depend on financial support by the public, and it is most appropriate to assume that their decisions which species to support and which not to is driven by similar factors. Therefore, a deeper understanding of the factors that affect various aspects of the WZC is very important.

It was previously demonstrated that large species selectively attract human attention and conservation efforts [85], [86]. We confirmed that the body size affects fundamentally all components of mammalian representation in the WZC. Mammalian species characterized by a large body size have a higher probability to be included in WZC. They tend to be represented by more numerous populations and they are also kept by more zoos. Because the material cost of keeping animals increases with the body size of the animal (Balmford 1996), the preferential representation of large mammals in the WZC is an interesting phenomenon. The metabolism (and thus the amounts of food and feces) and the required area of enclosure exponentially increase with the body size to about ¾ (0.72; [87], [88]; for review see [89]) and 2/3 (i.e., the length of the suggested enclosure is roughly proportional to the body length of the animal in breeder's guidelines, e.g., [90]), respectively. The fact that keeping a large species is constrained by the available space and expenses is further illustrated by our results suggesting that the number of conspecifics per zoo tends to be smaller in species of a larger body size. However, it seems that the zoos are able to overcome these constraints and selectively keep large animals because these attract more visitors and thus secure countervailing income ([91]).

In our previous study, we performed a separate analysis of the WZC of selected mammalian clades and we demonstrated that human preferences affect the WZC positively in basal mammals and Laurasiatheria [17]. The results of this study first show this relationship across all mammalian families; the species belonging to aesthetically attractive families have a higher probability to be included in the WZC, and they tend to be represented by more numerous populations as well as being kept by more zoos.

The positive effect of the encephalization index on the representation of mammalian species in the WZC predicted by [47] was only confirmed in the case of the proportion of zoo species. Apparently species of small-brained clades have a higher probability to be entirely omitted by the world zoos and their managers and curators. In agreement with Portman, the overall brain size (non-human primates: [92]), relative brain size (executive brain size: [93]; residuals: [94]) or the encephalization quotient, EQ = E_a_/E_e_, indicate the extent to which the brain size of a particular species E_a_ deviates from the expected brain size E_e_, and are, to some extent, good predictors of so-called “intelligence” of a mammal (for a review, see [95]).

However, the exact definition of the term “intelligence”, or higher cognitive abilities, is ambiguous. There are two main conflicting views: the adaptive specializations theory [96], saying that “intelligence” includes various learning and memory processes, which lead to adaptations for specific ecological task resolutions; and the general process view of “intelligence”, described by the existence of general associative-learning abilities, which differ quantitatively among species [97]. A new, consensual theory describes “intelligence” as a behavioral flexibility [95] manifested in quick problem-solving task or number of innovations [94]. Fagen [98] assumed that the number of innovations and play has close or causal relationship. In mammals, the large-brained taxa are more likely to contain species that play more often [99], and playful and active animals are more attractive to zoo visitors (e.g., Felidae: [100]). The attractiveness of animals with interesting behavior was also confirmed in [60], which reported higher willingness to support birds with special behavioral characteristics, e.g., courtship rituals.

Species richness of a family has a negative effect on the proportion of zoo species, the mean population size, and the number of conspecifics per zoo. Thus, the zoo curators tend to avoid simultaneous keeping of species belonging to the same family. This can be explained by human tendency to categorize mammals into primary cognitive categories frequently joining multiple scientific species into a single unit. Ethnobiologists repeatedly demonstrated that the primary units of human categorization of animals correspond to so-called “generic names” (for a review see [48]). These are one-word terms describing a typical species of a “genus”; additional species names are derived by adding the adjectives.

The knowledge of the factors affecting the selection of animals in the WZC might be applicable to a broad array of efforts influenced by human-induced selection of species. Once we become aware of it, we may adjust future planning of conservation projects to lead them into a better success, saving both time and finances along the way. Zoo curators may intentionally try to select unattractive, but needful, endangered species along with the attractive ones to be included in their collection to fulfill both the advisable ex-situ conservation role and the expectation of zoo visitors. For example, the least attractive mammalian family of the marsupial moles (Notoryctidae) includes only two species, both endangered according to the IUCN status. They are not kept in any zoo at present, and their future is very insecure unless selectively focused on, going against the unwanted bias. Another way to fulfill both of the roles is to select the most attractive species out of a list of species with a similar threat status. For example, there are both attractive and unattractive species within some animal families, as shown by Frynta et al. [43], on all 367 parrots of the family Psittacidae. From within such families, endangered yet attractive animals could be included in the WZC.

Whatever the conservation priorities are ([101]), if there is an existing tendency to prefer the conservation of some species over the others, the factors affecting this tendency should be known. Nowadays, the preservation of biodiversity is a widely accepted priority for species conservation (e.g., [102], [103]). This priority is in conflict with human tendency to pay the proper attention selectively to large and attractive animals. However, the awareness of this selectivity may help the conservationists to improve their strategies. There are many small and/or unattractive species that are phylogenetically significant and thus key for biodiversity preservation. With only little or no support received, these species could be lost forever. A good example of possible application of species attractiveness assessment is in the case of the EDGE species (Evolutionarily Distinct and Globally Endangered [104]) – a selection of threatened species with high biodiversity value. Once known, the unattractive species putatively lacking public awareness and support could receive special attention by conservation specialists.

Moreover, knowing that the attractiveness of an animal itself plays a major role in a human's decision-making, they may be able to intentionally select the “beautiful“ species not only to raise the zoo's popularity among visitors, but also to use such species in educational programs, or present them as flagship species for further in-situ protection of wildlife. A reasonable definition of flagship species was proposed by Verissimo et al. [105] (page 2): “A species used as the focus of a broader conservation marketing campaign based on its possession of one or more traits that appeal to the target audience”. Smith et al. [106] found that large bodied mammalian species with forward-facing eyes are most frequently used as flagships by non-government conservational organizations (NGO) and, based on these characteristics, they suggested five critically endangered species with a strong potential to serve as good flagship species: the African wild ass, tamarau (dwarf buffalo), pygmy raccoon, Talaud bear cuscus and Pennant's red colobus. When compared with our results, four of these animals belong to attractive mammalian families positioned in the top-half rank of the attractiveness (up to the fifth position; Equidae 7, Bovidae 20, Procyonidae 38 and Phalangeridae 50). The high attractiveness of threatened species per se may further increase the potential of these or similarly selected species to serve as flagships. In contrast, the Pennant's red colobus belongs to the family Cercopithecidae which appeared to be rather unattractive (placing the 94^th^ rank position). However, this family contains a large number of species the attractiveness of which may vary. A more detailed analysis on a species level could help to determine the actual attractiveness of the red colobus, or help find a species with similar attributes but higher attractiveness to be used as a flagship instead.

A properly selected flagship species may convince the public to donate more money for conservation, just as demonstrated by [107]. In their study, the respondents were willing to pay more for the conservation of an otter than that of a water vole. This is in accord with our finding that otters from the family Mustelidae are more attractive to humans, placing 34^th^ in the preference ranking, than water voles from the family Muridae, which placed as far as 73^rd^ (See Appendix S1). Also, when lumped together into one conservation program, these two animals received less support than otters alone [107]. This may be explained either by the sole presence of the unattractive animal in the program, which pushes the respondents back, or by the rising complexity of the message that was presented to the respondents. Either way, if conservationists select a single highly attractive animal to be presented to the public as a messenger for conservation planning, it may raise the success of the project. This may be caused both by raising the financial support of the project by people living far away from the place of question, or by local people who may re-think their view of the natural riches surrounding them [37]. Furthermore, we found that the attractiveness of snakes as perceived by humans is shared among such different cultures as Europeans and villagers from Papua New Guinea [62]. Another study confirmed these results on people from the five main inhabited continents [63]. If applicable to other animal taxa, the message from a single flagship species could touch people worldwide as well as people local to the conservation project. Although local communities face various problems with potential flagships that trigger conflicts as predatory animals, competitors or pests ([108], [109]), the attractiveness may play its role when the flagship is selected from harmless, non-conflicting species. In case of highly attractive animals, the attractiveness may even outweigh the possible conflict. The family Equidae placed 7^th^ rank of attractiveness in our study and the reintroduction of the Przewalski horse was well-accepted by local people in Mongolia (Hustain Nuru; Kůs E., Zoo Prague, personal communication) despite it being a competitor for domestic horses ([26]).

In conclusion, this study shows that the predictors associated with human attention, especially body size and aesthetic attractiveness, have a substantial effect on the composition of the WZC. In the 21^st^ century, it is of an utmost importance to pay attention to the biodiversity preservation, and it might be up to the worldwide zoological gardens to play a significant role in this task. This is especially because zoos have the capacity to hold numerous species, a capacity larger than any other institution, together with the knowledge about the breeding of various species and properly managed studbooks. Whether they utilize this potential is a vision of the future. However, for the zoos and conservationists whose intentions are to conserve biodiversity, our study reveals one of many factors – the human factor – that may fundamentally affect the conservation efforts. Thus, conservation biologists should consider these psychological factors for proper management of the “Ark”.

## Supporting Information

Appendix S1 The dataset used for statistical analyses, sorted by attractiveness from the most attractive family to the least attractive one.
(See under the Materials and methods section for the definition of the variables.)
(XLS)

Appendix S2 The forms and species listed in the ISIS database that were excluded from (or added to) the analysis.
(PDF)

Appendix S3 The literary sources of brain size data.
(PDF)

Appendix S4 Review of mammals reintroduced during the years 1992–2009. We collected journals and book sources of mammalian reintroductions within the years 1992–2009 with detailed information about the reintroduction events.
(PDF)

## References

[1] Sanderson EW, Jaiteh M, Levy MA, Redford KH, Wannebo AV, et al (2002) The human footprint and the last of the wild. Bio Science 52: 891–904.

[2] Brooks TM, Mittermeier RA, da Fonseca GAB, Gerlach J, Hoffmann M, et al (2006) Global biodiversity conservation priorities. Science 313: 58–61. 16825561 10.1126/science.1127609

[3] Wilson EO (2002) The future of life. New York, NY: Alfred A. Knopf.

[4] Marton-Lefèvre J (2010) Biodiversity is our life. Science 327: 1179. 20203016 10.1126/science.1188424

[5] Soulé M, Gilpin M, Conway W, Foose T (1986) The millenium Ark: How long a voyage, how many staterooms, how many passengers? Zoo Biol 5: 101–113.

[6] McGregor Reid G, Zippel KC (2008) Can zoos and aquariums ensure the survival of amphibians in the 21st century? Int Zoo Yearb 42: 1–6.

[7] Rees PA (2005) Will the EC Zoos Directive increase the conservation value of zoo research? Oryx 39: 128–131.

[8] Gippoliti S (2012) Ex situ conservation programmes in European zoological gardens: Can we afford to lose them? Biodivers Conserv 21: 1359–1364.

[9] Ebenhard T (1995) Conservation breeding as a tool for saving snímal species from extinction. Trends Ecol Evol 10: 438–443. 21237098 10.1016/s0169-5347(00)89176-4

[10] Stanley Price MR, Soorae PS (2003) Reintroductions: whence and whither? Int Zoo Yearb 38: 61–75.

[11] Bowkett AE (2009) Recent captive-breeding proposals and the return of the ark concept to global species conservation. Conserv Biol 23: 773–776. 19220367 10.1111/j.1523-1739.2008.01157.x

[12] Soulé ME (1980) Thresholds for survival: maintaining fitness and evolutionary potential. In: Soulé ME, Wilcox BA, editors. Conservation Biology: An Evolutionary-ecological Perspective. Sunderland, MA: Sinauer. pp. 151–169.

[13] Reed DH, Nicholas AC, Stratton GE (2007) Genetic quality of individuals impacts population dynamics. Anim Conserv 10: 275–283.

[14] Kimura M (1983) The neutral theory of molecular evolution. Cambridge, MA: Cambridge University Press.

[15] Lande R (1999) Extinction risks from anthropogenic, ecological, and genetics factors. In: Landweber LF, Dobson AP, editors. Genetics and the extinction of species: DNA and the conservation of biodiversity. Princeton, NJ: Princeton University Press. pp. 1–23.

[16] Frankham R, Ballou JD, Briscoe DA (2002) Introduction to conservation genetics. Cambridge, UK: Cambridge University Press.

[17] Frynta D, Marešová J, Landová E, Lišková S, Šimková O, et al.. (2009) Are animals in zoos rather conspicuous than endangered? In: Columbus AM, Kuznetsov L, editors. Endangered species: new research. New York, NY: Nova Science Publishers. pp. 299–341.

[18] Conde DA, Flesness N, Colchero F, Jones OR, Scheuerlein A (2011) An emerging role of zoos to conserve biodiversity. Science 331: 1390–1391. 21415339 10.1126/science.1200674

[19] Hale KA, Briskie JV (2007) Challenges to understanding the consequences of population bottlenecks for the conservation of endangered wildlife. Anim Conserv 10: 19–21.

[20] Roberts L (1988) Beyond Noah's Ark: what do we need to know? Science 242: 1247. 17817070 10.1126/science.242.4883.1247

[21] Snyder NFR, Derrickson SR, Beissinger SR, Wiley JW, Smith TB, et al (1996) Limitations of captive breeding in endangered species recovery. Conserv Biol 10: 338–348.

[22] Fischer J, Lindenmayer DB (2000) An assessment of the published results of animal relocations. Biol Conserv 96: 1–11.

[23] Mathews F, Orros M, McLaren G, Gelling M, Foster R (2005) Keeping fit on the ark: assessing the suitability of captive-bred animals for release. Biol Conserv 121: 569–577.

[24] Lees CM, Wilcken J (2009) Sustaining the Ark: the challenges faced by zoos in maintaining viable populations. Int Zoo Yearb 43: 6–18.

[25] Earnhardt JM (2010) The role of captive populations in reintroduction programs. In: Kleiman DG, Thompson KV, Baer CK, editors. Wild Mammals In Captivity. Chicago: University of Chicago Press. pp. 268–280.

[26] Volf J (2009) Half a century of international cooperation in the preservation of the Przewalski Horse - direction: reintroduction. In: Anonymous, editor. Equus 2009. Prague, CZ: Zoo. pp. 39–56.

[27] Freese CH, Aune KE, Boyd DP, Derr JN, Forrest SC, et al (2007) Second chance for the plains bison. Biol Conserv 136: 175–184.

[28] Ahrens TG (1921) The present status of the European bison or wisent. J Mammal 2: 58–62.

[29] Pucek Z, editor (2002) European bison: current state of the species and an action plan for its conservation. Species Action Plan, LHI-WWF.

[30] Tokarska M, Kawałko A, Wójcik JM, Pertoldi C (2009) Genetic variability in the European bison (*Bison bonasus*) population from Białowieża forest over 50 years. Bio J Linn Soc 97: 801–809.

[31] Jiang Z, Yu C, Feng Z, Zhang L, Xia J, et al (2000) Reintroduction and recovery of Père David's deer in China. Wildlife Soc B 28: 681–687.

[32] Stanley Price MR (1989) Animal re-introductions, the Arabian Oryx in Oman. Cambridge, UK: Cambridge University.

[33] Saltz D, Rubenstein DI (1995) Population dynamics of a reintroduced Asiatic wild ass (*Equus Hemionus*) herd. Ecol Appl 5: 327–335.

[34] Stoinski TS, Beck BB (2004) Changes in locomotor and foraging skills in captive-born, reintroduced golden lion tamarins (*Leontopithecus rosalia rosalia*). Am J Primatol 62: 1–13. 14752809 10.1002/ajp.20002

[35] Dobson A, Lyles A (2000) Black-footed ferret recovery. Science 288: 985–988. 10841720 10.1126/science.288.5468.985

[36] Leader-Williams N, Balmford A, Linkie M, Mace GM, Smith RJ, et al.. (2007) Beyond the ark: conservation biologists' views of the achievements of zoos in conservation. In: Zimmermann A, Hatchwell M, Dickie L, West C, editors. Zoos in the 21st Century: Catalysts for Conservation? Cambridge, UK: Cambridge University Press. pp. 236–254.

[37] Dietz JM, Dietz LA, Nagagata EY (1994) The effective use of flagship species for conservation of biodiversity: the example of lion tamarins in Brazil. In: Olney PJS, Mace GM, Feistner ATC (eds) Creative conservation: interactive management of wild and captive animals. Chapman & Hall, London, pp 32–49.

[38] Zimmermann A (2010) The role of zoos in contributing to in situ conservation. In: Kleiman DG, Thompson KV, Baer CK, editors. Wild mammals in captivity. Chicago: University of Chicago Press. pp. 281–287.

[39] Mazur N, Clark TW (2000) Zoos and conservation: policy making and organizational challenges. Yale F&ES Bulletin 105: 1–17.

[40] Bitgood S, Patterson D (1987) Principles of exhibit design. Visitor Behavior 2: 4–6.

[41] Puan CL, Zakaria M (2007) Perception of visitors towards the role of zoos: a Malaysian perspective. Int Zoo Yearb 41: 226–232.

[42] Moss A, Esson M (2010) Visitor interest in zoo animals and the implications for collection planning and zoo education programmes. Zoo Biol 29: 715–731. 20333734 10.1002/zoo.20316

[43] Frynta D, Lišková S, Bültmann S, Burda H (2010) Being attractive brings advantages: the case of parrot species in captivity. PLoS ONE 5: e12568. 20830206 10.1371/journal.pone.0012568 PMC2935353

[44] Marešová J, Frynta D (2008) Noah's Ark is full of common species attractive to humans: The case of boid snakes in zoos. Ecol Econ 64: 554–558.

[45] Balmford A, Mace GM, Leader-Williams N (1996) Designing the Ark: setting priorities for captive breeding. Conserv Biol 10: 719–727.

[46] Ericson PG, Anderson CL, Britton T, Elzanowski A, Johansson US, et al (2006) Diversification of Neoaves: integration of molecular sequence data and fossils. Biol Letters 2: 543–547. 10.1098/rsbl.2006.0523 PMC1834003 17148284

[47] Portmann A (1979) Nové cesty biologie. In: Fiala J, Neubauer Z, Pinc Z, editors. Scientia & Philosophia 7 (1997). Praha: Katedra matematické logiky a filosofie matematiky, Matematicko-fyzikální fakulta UK.

[48] Berlin B (1992) Ethnobiological Classification: principles of categorization of plants and animals in traditional societies. Princeton, NJ: Princeton University Press.

[49] Wilson DE, Reeder DAM, editors (2005) Mammal species of the world: a taxonomic and geographic reference (3rd ed). Baltimore, MD: Johns Hopkins, University Press. Checklist available on: http://nmnhgoph.si.edu/msw/.

[50] Nowak RM (1999) Walker's mammals of the world. Baltimore, MD: The Johns Hopkins University Press.

[51] Jerison HJ (1955) Brain to body ratios and the evolution of intelligence. Science 121: 447–449. 14358669 10.1126/science.121.3144.447

[52] Jerison HJ (1963) Interpreting the evolution of the brain. Hum Biol 35: 263–291. 14063188

[53] Jerison HJ (1973) Evolution of the brain and intelligence. New York: Academic Press.

[54] Ashwell KWS (2008) Encephalization of Australian and New Guinean marsupials. Brain Behav Evol 71: 181–199. 18230970 10.1159/000114406

[55] Whitfield P, editor (1984) Longman illustrated animal encyclopedia. Harlow, Essex, England: Longman.

[56] Anděra M (1997–2000) Svět zvířat I-III. Savci 1–3. Praha, Albatros.

[57] Hutchins M, Kleiman DG, Geist V, McDade MC, editors (2004) Grzimek's animal life encyclopedia, 2nd edition. Volumes 12–16, Mammals I-V. Farmington Hills, MI: Gale Group.

[58] Myers P, Espinosa R, Parr CS, Jones T, Hammond GS, et al. (2013) The Animal Diversity Web. Available: http://animaldiversity.org.

[59] Central Intelligence Agency. “Country Comparison: Distribution of family income - Gini index“.cia.gov. Central Intelligence Agency, n.d. Web. 27 Mar. 2013. Available: https://www.cia.gov/library/publications/the-world-factbook/rankorder/2172rank.html.

[60] Veríssimo D, Fraser I, Groombridge J, Bristol R, MacMillan DC (2009) Birds as turism flagship species: a case study of tropical islands. Anim Conserv 12: 549–558.

[61] Schlegel J, Rupf R (2010) Attitudes towards potential animal flagship species in nature conservation: A survey among students of different educational institutions. J Nat Conserv 18: 278–290.

[62] Marešová J, Krása A, Frynta D (2009) We all appreciate the same animals: cross-cultural comparison of human aesthetic preferences for snake species in Papua New Guinea and Europe. Ethology 115: 297–300.

[63] Frynta D, Petrů M, Šklíba J, Šumbera R, Krása A, et al (2011) Crosscultural agreement in perception of animal beauty: Boid snakes viewed by people from three continents. Hum Ecol 39: 829–834.

[64] Cunningham MR, Roberts AR, Barbee AP, Druen PB, Wu C (1995) “Their ideas of beauty are, on the whole, the same as ours”: Consistency and variability in the cross-cultural perception of female physical attractiveness. J Pers Soc Psychol 68: 261–279.

[65] Entwistle AC, Stephenson PJ (2000) Small mammals and the conservation agenda. In: Entwistle A, Dunstone N, editors. Priorities for theConservation of Mammalian Diversity. Has the Panda had its Day? Cambridge, UK: Cambridge University Press. pp. 119–139.

[66] SPSS Inc. (2007) Spss, version 16.0. Available: http://www.winwrap.com.

[67] R Development Core Team (2010) R: A language and environment for statistical computing. R foundation for statistical computing, Austria: Vienna.

[68] Harvey PH, Pagel MD (1991) The comparative method in evolutionary biology. Oxford: Oxford University Press.

[69] Felsenstein J (1985) Phylogenies and the comparative method. Am Nat 125: 1–15.

[70] Bininda-Emonds ORP, Cardillo M, Jones KE, MacPhee RDE, Beck RMD, et al (2007) The delayed rise of present-day mammals. Nature 446: 507–512. 17392779 10.1038/nature05634

[71] Arnason U, Adegoke JA, Bodin K, Born EW, Esa YB, et al (2002) Mammalian mitogenomic relationships and the root of the Eutherian tree. P Natl Acad Sci U S A 99: 8151–8156. 10.1073/pnas.102164299 PMC123036 12034869

[72] Meredith RW, Westerman M, Springer MS (2009) A phylogeny of Diprotodontia (Marsupialia) based on sequences for five nuclear genes. Mol Phylogenet Evol 51: 554–571. 19249373 10.1016/j.ympev.2009.02.009

[73] Barros MC, Sampaio I, Schneider H (2008) Novel 12S mtDNA findings in sloths (Pilosa, Folivora) and anteaters (Pilosa, Vermilingua) suggest a true case of long branch attraction. Genet Mol Biol 31: 793–799.

[74] Agnarsson I, May-Collado LJ (2008) The phylogeny of Cetartiodactyla: The importance of dense taxon sampling, missing data, and the remarkable promise of cytochrome b to provide reliable species-level phylogenies. Mol Phylogenet Evol 48: 964–985. 18590827 10.1016/j.ympev.2008.05.046

[75] Dalerum F (2007) Phylogenetic reconstruction of carnivore social organizations. J Zool 273: 90–97.

[76] Flynn JJ, Finarelli JA, Zehr S, Hsu J, Nedbal MA (2005) Assessing the impact of increased sampling on resolving enigmatic relationships. Syst Biol 54: 317–337. 16012099 10.1080/10635150590923326

[77] Blanga-Kanfi S, Miranda H, Penn O, Pupko T, DeBry RW, et al (2009) Rodent phylogeny revised: analysis of six nuclear genes from all major rodent clades. BMC Evol Biol 9: 71. 19341461 10.1186/1471-2148-9-71 PMC2674048

[78] Huchon D, Douzery EJP (2001) From the Old World to the New World: a molecular chronicle of the phylogeny and biogeography of Hystricognath rodents. Mol Phylogenet Evol 20: 238–251. 11476632 10.1006/mpev.2001.0961

[79] Jansa SA, Giarlaa TC, Lim BK (2009) The phylogenetic position of the rodent genus Typhlomys and the geographic origin of Muroidea. J Mammal 90: 1083–1094.

[80] Jansa SA, Weksler M (2004) Phylogeny of muroid rodents: relationships within and among major lineages as determined by IRBP gene sequences. Mol Phylogenet Evol 31: 256–276. 15019624 10.1016/j.ympev.2003.07.002

[81] Chatterjee HJ, Ho SYW, Barnes I, Groves C (2009) Estimating the phylogeny and divergence times of primates using a supermatrix approach. BMC Evol Biol 9: 259. 19860891 10.1186/1471-2148-9-259 PMC2774700

[82] Martins EP (2001) COMPARE Version 4.4. Computer Programs for Statistical Analysis of Comparative Data. Available: http://compare.bio.indiana.edu.

[83] Garland T, Harvey PH, Ives AR (1992) Procedures for the analysis of comparative data using phylogenetically independent contrasts. Syst Biol 41: 18–32.

[84] StatSoft (2001) Statistica, version 6.0. Available: http://www.statsoft.com.

[85] Gunnthorsdottir A (2001) Physical attractiveness of an animal species as a decision factor for its preservation. Anthrozoos 14: 204–215.

[86] Metrick A, Weitzman ML (1996) Patterns of behavior in endangered species preservation. Land Econ 72: 1.

[87] Kleiber M (1932) Body size and metabolism. Hilgardia 6: 315–353.

[88] Kleiber M (1961) The fire of life: an introduction to animal energetics. New York: Wiley.

[89] Schmidt-Nielsen K (1984) Scaling: why is animal size so important? Cambridge, UK: Cambridge University Press.

[90] Anonymous (1996) Gutachten über Mindestanforderungen an die Haltung von Säudetieren. BMVEL Bonn

[91] Balmford A (2000) Separating fact from artifact in analyses of zoo visitor preferences. Conserv Biol 14: 1193–1195.

[92] Deaner RO, Isler K, Burkart J, van Schaik C (2007) Overall brain size, and not encephalization quotient, best predicts cognitive ability across non-human primates. Brain Behav Evol 70: 115–124. 17510549 10.1159/000102973

[93] Reader SM, Laland KN (2002) Social intelligence, innovation, and enhanced brain size in primates. P Natl Acad Sci U S A 99: 4436–4441. 10.1073/pnas.062041299 PMC123666 11891325

[94] Overington SE, Morand-Ferronb J, Boogerta NJ, Lefebvrea L (2009) Technical innovations drive the relationship between innovativeness and residual brain size in birds. Anim Behav 78: 1001–1010.

[95] Roth G, Dicke U (2005) Evolution of the brain and intelligence. Trends Cogn Sci 9: 250–257. 15866152 10.1016/j.tics.2005.03.005

[96] Shettleworth SJ (2003) Memory and hippocampal specialization in food-storing birds: challenges for research on comparative cognition. Brain Behav Evol 62: 108–116. 12937349 10.1159/000072441

[97] Macphail EM, Bolhuis JJ (2001) The evolution of intelligence: adaptive specializations versus general process. Biol rev Camb Philos 76: 341–364. 10.1017/s146479310100570x 11569788

[98] Fagen R (1974) Selective and evolutionary aspects of animal play. Am Nat 108: 850–858.

[99] Iwaniuk AN, Nelson JE, Pellis SM (2001) Do big-brained animals play more? Comparative analyses of play and relative brain size in mammals. J Comp Psychol 115: 29–41. 11334216 10.1037/0735-7036.115.1.29

[100] Margulis SW, Hoyos C, Anderson M (2003) Effect of felid activity on zoo visitor interest. Zoo Biol 22: 587–599.

[101] Callicott JB (1990) Whither conservation ethics? Conserv Biol 4: 15–20.

[102] Margules CR, Pressey RL (2000) Systematic conservation planning. Nature 405: 243–253. 10821285 10.1038/35012251

[103] Smith RJ, Veríssimo D, MacMillan DC (2011) Marketing and conservation: how to lose friends and in fluence people. In: Leader-Williams N, Adams W, Smith R, editors. Trade-Offs in conservation: deciding what to save. pp. 215–232. Oxford, UK: Wiley Blackwell.

[104] Isaac NJ, Turvey ST, Collen B, Waterman C, Baillie JE (2007) Mammals on the EDGE: conservation priorities based on threat and phylogeny. PLoS ONE 2: e296. 17375184 10.1371/journal.pone.0000296 PMC1808424

[105] Verissimo D, MacMillan DC, Smith RJ (2011) Toward a systematic approach for identifying conservation flagships. Conserv Lett 4: 1–8.

[106] Smith RJ, Veríssimo D, Isaac NJB, Jones KE (2012) Identifying Cinderella species: uncovering mammals with conservation flagship appeal. Conserv Lett 5: 205–212.

[107] White PCL, Gregory KW, Lindley PJ, Richards G (1997) Economic values of threatened mammals in Britain: A case study of the otter *Lutra lutra* and the watervole *Arvicola terrestris* . Biol Conserv 82: 345–354.

[108] Kaltenborn BP, Bjerke T, Nyahongo JW, Williams DR (2006) Animal preferences and acceptability of wildlife management actions around Serengeti National Park, Tanzania. Biodivers Conserv 15: 4633–4649.

[109] Bowen-Jones E, Entwistle A (2002) Identifying appropriate flagship species: the importance of culture and local contexts. Oryx 36: 189–195.

