# Supplementary material for: Mammalian Collection on Noah's Ark: The Effects of Beauty, Brain and Body Size
Source: PLoS One. 2013 May 15;8(5):e63110. doi: 10.1371/journal.pone.0063110 (PMC3654911; doi:10.1371/journal.pone.0063110)
Supplement: Appendix S2 — The forms and species listed in the ISIS database that were excluded from (or added to) the analysis. (PDF) [file pone.0063110.s002.pdf]

Appendix 2. The forms and species listed in the ISIS database that were excluded from (or added to) the analysis.

Domestic species and species containing domestic forms from the ISIS database.

Family	Name from ISIS	Name for analysis
Bovidae	Gayal or gaur, *Bos frontalis*	omitted
	Domestic gayal, *Bos f. frontalis*	omitted
	Gaur, *Bos frontalis gaurus*	*Bos frontalis*
	Yak, *Bos grunniens*	omitted
	Domestic yak, *Bos g. grunniens*	omitted
	Domestic yak, *Bos g. grunniens domestic*	omitted
	Wild yak, *Bos g. mutus*	*Bos grunniens*
	Cow or ox, *Bos taurus* ssp.	omitted
	Banteng, *Bos javanicus*	*Bos javanicus*
	Javan banteng, *Bos j. javanicus*	*Bos javanicus*
	Sheep, *Ovis aries* , *O. a. aries*	omitted
	Mouflon, *Ovis aries musimon*	*Ovis aries* *
	Goat, *Capra hircus* , *C. h. hircus* sssp	omitted
	Western wild goat, *Capra hircus aegagrus* , *C. h. blythi*	*Capra hircus*
	Cretan wild goat, *Capra hircus cretica*	*Capra hircus*
Camelidae	Domestic bactrian camel, *Camelus bactrianus domestic*	omitted
	Domestic dromedary camel, *Camelus dromedarius domestic*	omitted
	Llama, *Lama glama*	omitted
	Guanaco, *Lama guanicoe*	*Lama glama*
	Alpaca, *Lama pacos*	omitted
	Domestic alpaca, *Lama pacos domestic*	omitted
Suidae	Wild boar, *Sus scrofa scrofa* , *S. s. taivanus* , *S. s. vittatus*	*Sus scrofa*
	Domestic pig, *Sus scrofa scrofa domestic* , *S. s. s.* sssp	omitted
Equidae	African wild ass, *Equus asinus*	*Equus asinus*
	Nubian wild ass, *Equus asinus africanus*	*Equus asinus*
	Domestic ass or donkey, *Equus asinus asinus* sssp	omitted
	Somali wild ass, *Equus asinus somalicus*	*Equus asinus*
	Horse, *Equus caballus*	omitted
	Domestic horse, *E. c. caballus*	omitted
	Przewalski's wild horse, *E. c. przewalskii*	*Equus caballus*
Caviidae	Domestic guinea pig, *Cavia porcellus*	omitted
Felidae	Domestic cat, *Felis catus*	omitted
Canidae	Domestic dog, *Canis lupus familiaris*	omitted
	Dingo, *Canis lupus dingo*	*Canis dingo* *
Mustelidae	Domestic polecat, *Mustela putorius furo*	omitted
Muridae	Gold hamster, *Mesocricetus auratus*	omitted

* ancient feral population, living without human care for very long time

Frynta, D. et al. 2012. Mammalian collection on Noah’s Ark: the effects of beauty, brain and body size.
