# Supplementary material for: Mammalian Collection on Noah's Ark: The Effects of Beauty, Brain and Body Size
Source: PLoS One. 2013 May 15;8(5):e63110. doi: 10.1371/journal.pone.0063110 (PMC3654911; doi:10.1371/journal.pone.0063110)
Supplement: Appendix S3 — The literary sources of brain size data. (PDF) [file pone.0063110.s003.pdf]

Appendix 3. The literary sources of brain size data.

| Family | Number of species | Source |
|---|---|---|
| Acrobatidae | 2 | Ashwell, K.W.S. 2008. Encephalization of Australian and New Guinean Marsupials. Brain. Behav. Evol. 71:181–199. |
| Ailuridae | 1 | Dunbar, R. I. M. and Bever, J. 1998. Neocortex size predicts group size in carnivores and some insectivores. Ethology 104: 695–708.<br>Pérez-Barbería, F. J., Shultz, S. and Dunbar, R. I. M. 2007. Evidence for coevolution of sociality and relative brain size in three orders of mammals. Evolution 61: 2811–2821. |
| Antilocapridae | 1 | Pérez-Barbería, F. J., Shultz, S. and Dunbar, R. I. M. 2007. Evidence for coevolution of sociality and relative brain size in three orders of mammals. Evolution 61: 2811–2821. |
| Aotidae | 1 | Bauchot, R. and Stephan, H. 1969. Encephalisation et niveau evolutif chez les simiens. Mammalia 33: 225–275.<br>Bronson, R. T. 1981. Brain weight-body weight relationships in 12 species of nonhuman primates. Am. J. Phys. Anthropol. 56:77–81.<br>Stephan, H., Frahm, H. and Baron, G. 1981. New and revisited data on volumes of brain structures in Insectivores and Primates. Folia Primatol. 35: 1–29.<br>Marino, L. 1998. A Comparison of encephalization between Odontocete Cetaceans and Anthropoid Primates. Brain. Behav. Evol. 51: 230–238.<br>Stephan, H., Baron, G. and Frahm, H. D. 1998. Comparative size of brains and brain components. In: Steklis, H. D. and Erwin, J. (eds), Comparative primate biology, vol. 4: Neurosciences. pp 1–38, Alan R. Liss, Inc., New York.<br>Herculano-Houzel, S., Collins, C. E., Wong, P. and Kaas, J. K. 2007. Cellular scaling rules for primate brains. PNAS 104:3562–3567.<br>Pérez-Barbería, F. J., Shultz, S. and Dunbar, R. I. M. 2007. Evidence for coevolution of sociality and relative brain size in three orders of mammals. Evolution 61: 2811–2821. |
| Aplodontiidae | 1 | Mace, G. M., Harvey, P. H. and Clutton-Brock T. H. 1981. Brain size and ecology in small mammals. J. Zool. Lond. 193: 333–354.<br>McNab, B. K. and Eisenberg, J. F. 1989. Brain size and its relation to the rate of metabolism in mammals. Am. Nat. 133: 157–167. |
| Atelidae | 8 | Hrdlička, A. 1925. Weight of the brain and of the internal organs in american monkeys. Am. J. Phys. Anthropol. 8:201–211.<br>Crile, G. M. D. and Quiring, D. P. 1940. A record of the body weight and certain organ and gland weights of 3690 animals. Ohio J. Sci. 5: 219–259.<br>Bauchot, R. and Stephan, H. 1969. Encephalisation et niveau evolutif chez les simiens. Mammalia 33: 225–275.<br>Sacher, G. A. and Staffeldt, E. F. 1974. Relation of gestation time to brain weight for placental mammals: implications for the theory of vertebrate growth. Am. Nat. 108: 593–615.<br>Stephan, H., Frahm, H. and Baron, G. 1981. New and revisited data on volumes of brain structures in Insectivores and Primates. Folia Primatol. 35: 1–29. |

| | | |
|---|---|---|
| | | Bronson, R. T. 1981. Brain weight-body weight relationships in 12 species of nonhuman primates. Am. J. Phys. Anthropol. 56: 77–81.<br>Stephan, H., Baron, G. and Frahm, H. D. 1998. Comparative size of brains and brain components. In: Steklis, H. D. and Erwin, J. (eds), Comparative primate biology, vol. 4: Neurosciences. pp 1–38, Alan R. Liss, Inc., New York.<br>Marino, L. 1998. A Comparison of encephalization between Odontocete Cetaceans and Anthropoid Primates. Brain. Behav. Evol. 51: 230–238.<br>Pérez-Barbería, F. J., Shultz, S. and Dunbar, R. I. M. 2007. Evidence for coevolution of sociality and relative brain size in three orders of mammals. Evolution 61: 2811–2821. |
| Bathyergidae | 4 | Mace, G. M., Harvey, P. H. and Clutton-Brock T. H. 1981. Brain size and ecology in small mammals. J. Zool. Lond. 193: 333–354.<br>McNab, B. K. and Eisenberg, J. F. 1989. Brain size and its relation to the rate of metabolism in mammals. Am. Nat. 133: 157–167. |
| Bovidae | 45 | Sacher, G. A. and Staffeldt, E. F. 1974. Relation of gestation time to brain weight for placental mammals: implications for the theory of vertebrate growth. Am. Nat. 108: 593–615.<br>McNab, B. K. and Eisenberg, J. F. 1989. Brain size and its relation to the rate of metabolism in mammals. Am. Nat. 133: 157–167.<br>Pérez-Barbería, F. J., Shultz, S. and Dunbar, R. I. M. 2007. Evidence for coevolution of sociality and relative brain size in three orders of mammals. Evolution 61: 2811–2821. |
| Bradypodidae | 1 | Crile, G. M. D. and Quiring, D. P. 1940. A record of the body weight and certain organ and gland weights of 3690 animals. Ohio J. Sci. 5: 219–259.<br>McNab, B. K. and Eisenberg, J. F. 1989. Brain size and its relation to the rate of metabolism in mammals. Am. Nat. 133: 157–167. |
| Burramyidae | 5 | Ashwell, K.W.S. 2008. Encephalization of Australian and New Guinean Marsupials. Brain. Behav. Evol. 71:181–199. |
| Caenolestidae | 2 | Ashwell, K.W.S. 2008. Encephalization of Australian and New Guinean Marsupials. Brain. Behav. Evol. 71:181–199. |
| Camelidae | 4 | Sacher, G. A. and Staffeldt, E. F. 1974. Relation of gestation time to brain weight for placental mammals: implications for the theory of vertebrate growth. Am. Nat. 108: 593–615.<br>McNab, B. K. and Eisenberg, J. F. 1989. Brain size and its relation to the rate of metabolism in mammals. Am. Nat. 133: 157–167.<br>Pérez-Barbería, F. J., Shultz, S. and Dunbar, R. I. M. 2007. Evidence for coevolution of sociality and relative brain size in three orders of mammals. Evolution 61: 2811–2821. |
| Canidae | 24 | Sacher, G. A. and Staffeldt, E. F. 1974. Relation of gestation time to brain weight for placental mammals: implications for the theory of vertebrate growth. Am. Nat. 108: 593–615.<br>Gittleman, J. L. 1986. Carnivore brain size, behavioral ecology, and phylogeny. J. Mammal. 67: 23–36.<br>McNab, B. K. and Eisenberg, J. F. 1989. Brain size and its relation to the rate of metabolism in mammals. Am. Nat. 133: 157–167.<br>Pérez-Barbería, F. J., Shultz, S. and Dunbar, R. I. M. 2007. Evidence for coevolution of sociality and relative brain size in three orders of mammals. Evolution 61: 2811–2821. |

| | | |
|---|---|---|
| Capromyidae | 1 | Mace, G. M., Harvey, P. H. and Clutton-Brock T. H. 1981. Brain size and ecology in small mammals. J. Zool. Lond. 193: 333–354 |
| Castoridae | 2 | Sacher, G. A. and Staffeldt, E. F. 1974. Relation of gestation time to brain weight for placental mammals: implications for the theory of vertebrate growth. Am. Nat. 108: 593–615. |
| Caviidae | 3 | Sacher, G. A. and Staffeldt, E. F. 1974. Relation of gestation time to brain weight for placental mammals: implications for the theory of vertebrate growth. Am. Nat. 108: 593–615.<br>Mace, G. M., Harvey, P. H. and Clutton-Brock T. H. 1981. Brain size and ecology in small mammals. J. Zool. Lond. 193: 333–354.<br>McNab, B. K. and Eisenberg, J. F. 1989. Brain size and its relation to the rate of metabolism in mammals. Am. Nat. 133: 157–167. |
| Cebidae | 13 | Hrdlička, A. 1905. Brain weight in vertebrates. Smithsonian Miscellaneous Collections 48:89–112.<br>Hrdlička, A. 1925. Weight of the brain and of the internal organs in american monkeys. Am. J. Phys. Anthropol. 8:201–211.<br>Crile, G. M. D. and Quiring, D. P. 1940. A record of the body weight and certain organ and gland weights of 3690 animals. Ohio J. Sci. 5: 219–259.<br>Bauchot, R. and Stephan, H. 1966. Donnees nouvelles sur l'encephalization des insectivores et des Prosimiens. Mammalia 30: 160–196.<br>Bauchot, R. and Stephan, H. 1969. Encephalisation et niveau evolutif chez les simiens. Mammalia 33: 225–275.<br>Sacher, G. A. and Staffeldt, E. F. 1974. Relation of gestation time to brain weight for placental mammals: implications for the theory of vertebrate growth. Am. Nat. 108: 593–615.<br>Stephan, H., Frahm, H. and Baron, G. 1981. New and revisited data on volumes of brain structures in Insectivores and Primates. Folia Primatol. 35: 1–29.<br>Bronson, R. T. 1981. Brain weight-body weight relationships in 12 species of nonhuman primates. Am. J. Phys. Anthropol. 56: 77–81.<br>Marino, L. 1998. A Comparison of encephalization between Odontocete Cetaceans and Anthropoid Primates. Brain. Behav. Evol. 51: 230–238.<br>Stephan, H., Baron, G. and Frahm, H. D. 1998. Comparative size of brains and brain components. In: Steklis, H. D. and Erwin, J. (eds), Comparative primate biology, vol. 4: Neurosciences. pp 1–38, Alan R. Liss, Inc., New York.<br>Herculano-Houzel, S., Collins, C. E., Wong, P. and Kaas, J. K. 2007. Cellular scaling rules for primate brains. PNAS 104:3562–3567.<br>Pérez-Barbería, F. J., Shultz, S. and Dunbar, R. I. M. 2007. Evidence for coevolution of sociality and relative brain size in three orders of mammals. Evolution 61: 2811–2821. |
| Cercopithecidae | 41 | Hrdlička, A. 1925. Weight of the brain and of the internal organs in american monkeys. Am. J. Phys. Anthropol. 8:201–211.<br>Crile, G. M. D. and Quiring, D. P. 1940. A record of the body weight and certain organ and gland weights of 3690 animals. Ohio J. Sci. 5: 219–259.<br>Bauchot, R. and Stephan, H. 1969. Encephalisation et niveau evolutif chez les simiens. Mammalia 33: 225–275.<br>Sacher, G. A. and Staffeldt, E. F. 1974. Relation of gestation time to brain weight for placental mammals: implications for the theory of vertebrate growth. Am. Nat. 108: 593–615. |

| | | |
|---|---|---|
| | | Bronson, R. T. 1981. Brain weight-body weight relationships in 12 species of nonhuman primates. Am. J. Phys. Anthropol. 56: 77–81.<br>Stephan, H., Frahm, H. and Baron, G. 1981. New and revisited data on volumes of brain structures in Insectivores and Primates. Folia Primatol. 35: 1–29.<br>Stephan, H., Baron, G. and Frahm, H. D. 1998. Comparative size of brains and brain components. In: Steklis, H. D. and Erwin, J. (eds), Comparative primate biology, vol. 4: Neurosciences. pp 1–38, Alan R. Liss, Inc., New York.<br>Marino, L. 1998. A Comparison of encephalization between Odontocete Cetaceans and Anthropoid Primates. Brain. Behav. Evol. 51: 230–238.<br>Herculano-Houzel, S., Collins, C. E., Wong, P. and Kaas, J. K. 2007. Cellular scaling rules for primate brains. PNAS 104:3562–3567.<br>Pérez-Barbería, F. J., Shultz, S. and Dunbar, R. I. M. 2007. Evidence for coevolution of sociality and relative brain size in three orders of mammals. Evolution 61: 2811–2821. |
| Cervidae | 12 | Sacher, G. A. and Staffeldt, E. F. 1974. Relation of gestation time to brain weight for placental mammals: implications for the theory of vertebrate growth. Am. Nat. 108: 593–615.<br>McNab, B. K. and Eisenberg, J. F. 1989. Brain size and its relation to the rate of metabolism in mammals. Am. Nat. 133: 157–167.<br>Pérez-Barbería, F. J., Shultz, S. and Dunbar, R. I. M. 2007. Evidence for coevolution of sociality and relative brain size in three orders of mammals. Evolution 61: 2811–2821. |
| Cricetidae | 54 | Sacher, G. A. and Staffeldt, E. F. 1974. Relation of gestation time to brain weight for placental mammals: implications for the theory of vertebrate growth. Am. Nat. 108: 593–615.<br>Mace, G. M., Harvey, P. H. and Clutton-Brock T. H. 1981. Brain size and ecology in small mammals. J. Zool. Lond. 193: 333–354.<br>McNab, B. K. and Eisenberg, J. F. 1989. Brain size and its relation to the rate of metabolism in mammals. Am. Nat. 133: 157–167. |
| Ctenodactylidae | 2 | Mace, G. M., Harvey, P. H. and Clutton-Brock T. H. 1981. Brain size and ecology in small mammals. J. Zool. Lond. 193: 333–354 |
| Ctenomyidae | 29 | Vassallo, A. E. and Echeverría, A. I. 2009. Evolution of brain size in a highly diversifying lineage of subterranean rodent genus Ctenomys (Caviomorpha: Ctenomyidae). Brain. Behav. Evol. 73: 138–149. |
| Cuniculidae | 1 | McNab, B. K. and Eisenberg, J. F. 1989. Brain size and its relation to the rate of metabolism in mammals. Am. Nat. 133: 157–167. |
| Cyclopedidae | 1 | McNab, B. K. and Eisenberg, J. F. 1989. Brain size and its relation to the rate of metabolism in mammals. Am. Nat. 133: 157–167.<br>Hrdlička, A. 1905. Brain weight in vertebrates. Smithsonian Miscellaneous Collections 48:89–112.<br>Crile, G. M. D. and Quiring, D. P. 1940. A record of the body weight and certain organ and gland weights of 3690 animals. Ohio J. Sci. 5: 219–259. |
| Dasypodidae | 4 | Hrdlička, A. 1905. Brain weight in vertebrates. Smithsonian Miscellaneous Collections 48:89–112.<br>Crile, G. M. D. and Quiring, D. P. 1940. A record of the body weight and certain organ and gland weights of 3690 animals. Ohio J. Sci. 5: 219–259. |

| | | |
|---|---|---|
| | | Sacher, G. A. and Staffeldt, E. F. 1974. Relation of gestation time to brain weight for placental mammals: implications for the theory of vertebrate growth. Am. Nat. 108: 593–615.<br>McNab, B. K. and Eisenberg, J. F. 1989. Brain size and its relation to the rate of metabolism in mammals. Am. Nat. 133: 157–167. |
| Dasyproctidae | 2 | Sacher, G. A. and Staffeldt, E. F. 1974. Relation of gestation time to brain weight for placental mammals: implications for the theory of vertebrate growth. Am. Nat. 108: 593–615.<br>Mace, G. M., Harvey, P. H. and Clutton-Brock T. H. 1981. Brain size and ecology in small mammals. J. Zool. Lond. 193: 333–354.<br>McNab, B. K. and Eisenberg, J. F. 1989. Brain size and its relation to the rate of metabolism in mammals. Am. Nat. 133: 157–167. |
| Dasyuridae | 48 | McNab, B. K. and Eisenberg, J. F. 1989. Brain size and its relation to the rate of metabolism in mammals. Am. Nat. 133: 157–167.<br>Ashwell, K.W.S. 2008. Encephalization of Australian and New Guinean Marsupials. Brain. Behav. Evol. 71:181–199. |
| Daubentoniidae | 1 | Stephan, H., Frahm, H. and Baron, G. 1981. New and revisited data on volumes of brain structures in Insectivores and Primates. Folia Primatol. 35: 1–29.<br>Kaufman, J. A., Ahrens, E. T., Laidlaw, D. H., Zhang, S. and Allman, J. M. 2005. Anatomical analysis of an Aye-Aye brain (Daubentonia madagascariensis, Primates: Prosimii) combining histology, structural magnetic resonance imaging, and diffusion-tensor imaging. The Anatomical Record Part A 287: 1026–1037. |
| Didelphidae | 19 | Eisenberg, J. F. and Wilson, D. E. 1981. Relative brain size and demographic strategies in Didelphid Marsupials. Am. Nat. 118: 1–15.<br>McNab, B. K. and Eisenberg, J. F. 1989. Brain size and its relation to the rate of metabolism in mammals. Am. Nat. 133: 157–167.<br>Ashwell, K.W.S. 2008. Encephalization of Australian and New Guinean Marsupials. Brain. Behav. Evol. 71:181–199. |
| Dinomyidae | 1 | Mace, G. M., Harvey, P. H. and Clutton-Brock T. H. 1981. Brain size and ecology in small mammals. J. Zool. Lond. 193: 333–354. |
| Dipodidae | 5 | Mace, G. M., Harvey, P. H. and Clutton-Brock T. H. 1981. Brain size and ecology in small mammals. J. Zool. Lond. 193: 333–354.<br>McNab, B. K. and Eisenberg, J. F. 1989. Brain size and its relation to the rate of metabolism in mammals. Am. Nat. 133: 157–167. |
| Dugongidae | 1 | O'Shea, T. J. and Reep, R. L. 1990. Encephalization quotients and life-history traits in the Sirenia. J. mammal. 71: 534–543. |
| Echimyidae | 2 | Mace, G. M., Harvey, P. H. and Clutton-Brock T. H. 1981. Brain size and ecology in small mammals. J. Zool. Lond. 193: 333–354.<br>McNab, B. K. and Eisenberg, J. F. 1989. Brain size and its relation to the rate of metabolism in mammals. Am. Nat. 133: 157–167. |

| | | |
|---|---|---|
| Elephantidae | 1 | Sacher, G. A. and Staffeldt, E. F. 1974. Relation of gestation time to brain weight for placental mammals: implications for the theory of vertebrate growth. Am. Nat. 108: 593–615. |
| Equidae | 4 | Pérez-Barbería, F. J., Shultz, S. and Dunbar, R. I. M. 2007. Evidence for coevolution of sociality and relative brain size in three orders of mammals. Evolution 61: 2811–2821. |
| Erethizontidae | 1 | Sacher, G. A. and Staffeldt, E. F. 1974. Relation of gestation time to brain weight for placental mammals: implications for the theory of vertebrate growth. Am. Nat. 108: 593–615.<br>Mace, G. M., Harvey, P. H. and Clutton-Brock T. H. 1981. Brain size and ecology in small mammals. J. Zool. Lond. 193: 333–354. |
| Erinaceidae | 6 | Sacher, G. A. and Staffeldt, E. F. 1974. Relation of gestation time to brain weight for placental mammals: implications for the theory of vertebrate growth. Am. Nat. 108: 593–615.<br>Mace, G. M., Harvey, P. H. and Clutton-Brock T. H. 1981. Brain size and ecology in small mammals. J. Zool. Lond. 193: 333–354.<br>Stephan, H., Frahm, H. and Baron, G. 1981. New and revisited data on volumes of brain structures in Insectivores and Primates. Folia Primatol. 35: 1–29.<br>McNab, B. K. and Eisenberg, J. F. 1989. Brain size and its relation to the rate of metabolism in mammals. Am. Nat. 133: 157–167.<br>Stephan, H., Baron, G. and Frahm, H. D. 1991. Comparative brain research in mammals, volume 1: Insectivora. Springer-Verlag, New York. |
| Eupleridae | 5 | Gittleman, J. L. 1986. Carnivore brain size, behavioral ecology, and phylogeny. J. Mammal. 67:23–36.<br>Pérez-Barbería, F. J., Shultz, S. and Dunbar, R. I. M. 2007. Evidence for coevolution of sociality and relative brain size in three orders of mammals. Evolution 61: 2811–2821. |
| Felidae | 24 | Sacher, G. A. and Staffeldt, E. F. 1974. Relation of gestation time to brain weight for placental mammals: implications for the theory of vertebrate growth. Am. Nat. 108: 593–615.<br>Gittleman, J. L. 1986. Carnivore brain size, behavioral ecology, and phylogeny. J. Mammal. 67:23–36.<br>McNab, B. K. and Eisenberg, J. F. 1989. Brain size and its relation to the rate of metabolism in mammals. Am. Nat. 133: 157–167.<br>Pérez-Barbería, F. J., Shultz, S. and Dunbar, R. I. M. 2007. Evidence for coevolution of sociality and relative brain size in three orders of mammals. Evolution 61: 2811–2821. |
| Galagidae | 3 | Crile, G. M. D. and Quiring, D. P. 1940. A record of the body weight and certain organ and gland weights of 3690 animals. Ohio J. Sci. 5: 219–259.<br>Stephan, H., Frahm, H. and Baron, G. 1981. New and revisited data on volumes of brain structures in Insectivores and Primates. Folia Primatol. 35: 1–29.<br>Stephan, H., Baron, G. and Frahm, H. D. 1998. Comparative size of brains and brain components. In: Steklis, H. D. and Erwin, J. (eds), Comparative primate biology, vol. 4: Neurosciences. pp 1–38, Alan R. Liss, Inc., New York.<br>Pérez-Barbería, F. J., Shultz, S. and Dunbar, R. I. M. 2007. Evidence for coevolution of sociality and relative brain size in three orders of mammals. Evolution 61: 2811–2821. |
| Geomyidae | 10 | Mace, G. M., Harvey, P. H. and Clutton-Brock T. H. 1981. Brain size and ecology in small mammals. J. Zool. Lond. 193: 333–354. |

| | | |
|---|---|---|
| | | Hafner, M. S. and Hafner, J. C. 1984. Brain size, adaptation and heterochrony in Geomyoid Rodents. Evolution 38:1088–1098.<br>McNab, B. K. and Eisenberg, J. F. 1989. Brain size and its relation to the rate of metabolism in mammals. Am. Nat. 133: 157–167. |
| Giraffidae | 2 | Pérez-Barbería, F. J., Shultz, S. and Dunbar, R. I. M. 2007. Evidence for coevolution of sociality and relative brain size in three orders of mammals. Evolution 61: 2811–2821. |
| Herpestidae | 19 | Gittleman, J. L. 1986. Carnivore brain size, behavioral ecology, and phylogeny. J. Mammal. 67:23–36.<br>Röhrs, Von M., Ebinger, P., Weidemann, W. 1989. Cephalisation bei Viverridae, Hyaenidae, Procyonidae und Ursidae. Z. Zool. Syst. Evol. Forsch. 27:169–180.<br>Pérez-Barbería, F. J., Shultz, S. and Dunbar, R. I. M. 2007. Evidence for coevolution of sociality and relative brain size in three orders of mammals. Evolution 61: 2811–2821. |
| Heteromyidae | 24 | Mace, G. M., Harvey, P. H. and Clutton-Brock T. H. 1981. Brain size and ecology in small mammals. J. Zool. Lond. 193: 333–354.<br>Hafner, M. S. and Hafner, J. C. 1984. Brain size, adaptation and heterochrony in Geomyoid Rodents. Evolution 38:1088–1098.<br>McNab, B. K. and Eisenberg, J. F. 1989. Brain size and its relation to the rate of metabolism in mammals. Am. Nat. 133: 157–167. |
| Hippopotamidae | 2 | Sacher, G. A. and Staffeldt, E. F. 1974. Relation of gestation time to brain weight for placental mammals: implications for the theory of vertebrate growth. Am. Nat. 108: 593–615.<br>Pérez-Barbería, F. J., Shultz, S. and Dunbar, R. I. M. 2007. Evidence for coevolution of sociality and relative brain size in three orders of mammals. Evolution 61: 2811–2821. |
| Hominidae | 3 | Dubois, E. 1897. Sur le rapport du poids de l'encephale avec le grandeur du corps chez les mammifieres. Bull. Soc. Anthropol. Paris 8: 337–376.<br>Anthony, R. 1938. Essai de recherche d'une expression anatomique approximative du degré d'organisation cérébrale, autre que le poids de l'encéphale comparé au poids du corps. Bull. Soc. Anthropol. Paris 9:17–67.<br>Crile, G. M. D. and Quiring, D. P. 1940. A record of the body weight and certain organ and gland weights of 3690 animals. Ohio J. Sci. 5: 219–259.<br>Bauchot, R. and Stephan, H. 1969. Encephalisation et niveau evolutif chez les simiens. Mammalia 33: 225–275.<br>Sacher, G. A. and Staffeldt, E. F. 1974. Relation of gestation time to brain weight for placental mammals: implications for the theory of vertebrate growth. Am. Nat. 108: 593–615.<br>Stephan, H., Frahm, H. and Baron, G. 1981. New and revisited data on volumes of brain structures in Insectivores and Primates. Folia Primatol. 35: 1–29.<br>Marino, L. 1998. A Comparison of encephalization between Odontocete Cetaceans and Anthropoid Primates. Brain. Behav. Evol. 51: 230–238.<br>Stephan, H., Baron, G. and Frahm, H. D. 1998. Comparative size of brains and brain components. In: Steklis, H. D. and Erwin, J. (eds), Comparative primate biology, vol. 4: Neurosciences. pp 1–38, Alan R. Liss, Inc., New York.<br>Pérez-Barbería, F. J., Shultz, S. and Dunbar, R. I. M. 2007. Evidence for coevolution of sociality and relative brain size in three orders of mammals. Evolution 61: 2811–2821. |

| | | |
|---|---|---|
| Hyaenidae | 4 | McNab, B. K. and Eisenberg, J. F. 1989. Brain size and its relation to the rate of metabolism in mammals. Am. Nat. 133: 157–167.<br>Pérez-Barbería, F. J., Shultz, S. and Dunbar, R. I. M. 2007. Evidence for coevolution of sociality and relative brain size in three orders of mammals. Evolution 61: 2811–2821. |
| Hylobatidae | 5 | Dubois, E. 1897. Sur le rapport du poids de l'encephale avec le grandeur du corps chez les mammifieres. Bull. Soc. Anthropol. Paris 8: 337–376.<br>Hrdlička, A. 1925. Weight of the brain and of the internal organs in american monkeys. Am. J. Phys. Anthropol. 8:201–211.<br>Bauchot, R. and Stephan, H. 1969. Encephalisation et niveau evolutif chez les simiens. Mammalia 33: 225–275.<br>Sacher, G. A. and Staffeldt, E. F. 1974. Relation of gestation time to brain weight for placental mammals: implications for the theory of vertebrate growth. Am. Nat. 108: 593–615.<br>Stephan, H., Frahm, H. and Baron, G. 1981. New and revisited data on volumes of brain structures in Insectivores and Primates. Folia Primatol. 35: 1–29.<br>Marino, L. 1998. A Comparison of encephalization between Odontocete Cetaceans and Anthropoid Primates. Brain. Behav. Evol. 51: 230–238.<br>Stephan, H., Baron, G. and Frahm, H. D. 1998. Comparative size of brains and brain components. In: Steklis, H. D. and Erwin, J. (eds), Comparative primate biology, vol. 4: Neurosciences. pp 1–38, Alan R. Liss, Inc., New York.<br>Pérez-Barbería, F. J., Shultz, S. and Dunbar, R. I. M. 2007. Evidence for coevolution of sociality and relative brain size in three orders of mammals. Evolution 61: 2811–2821. |
| Hystricidae | 2 | Sacher, G. A. and Staffeldt, E. F. 1974. Relation of gestation time to brain weight for placental mammals: implications for the theory of vertebrate growth. Am. Nat. 108: 593–615.<br>Mace, G. M., Harvey, P. H. and Clutton-Brock T. H. 1981. Brain size and ecology in small mammals. J. Zool. Lond. 193: [illegible] |
| Cheirogaleidae | 3 | Bauchot, R. and Stephan, H. 1966. Donnees nouvelles sur l'encephalization des insectivores et des Prosimiens. Mammalia 30: 160–196.<br>Stephan, H., Frahm, H. and Baron, G. 1981. New and revisited data on volumes of brain structures in Insectivores and Primates. Folia Primatol. 35: 1–29.<br>Stephan, H., Baron, G. and Frahm, H. D. 1998. Comparative size of brains and brain components. In: Steklis, H. D. and Erwin, J. (eds), Comparative primate biology, vol. 4: Neurosciences. pp 1–38, Alan R. Liss, Inc., New York.<br>Pérez-Barbería, F. J., Shultz, S. and Dunbar, R. I. M. 2007. Evidence for coevolution of sociality and relative brain size in three orders of mammals. Evolution 61: 2811–2821. |
| Chinchillidae | 3 | Sacher, G. A. and Staffeldt, E. F. 1974. Relation of gestation time to brain weight for placental mammals: implications for the theory of vertebrate growth. Am. Nat. 108: 593–615.<br>Mace, G. M., Harvey, P. H. and Clutton-Brock T. H. 1981. Brain size and ecology in small mammals. J. Zool. Lond. 193: 333–354.<br>McNab, B. K. and Eisenberg, J. F. 1989. Brain size and its relation to the rate of metabolism in mammals. Am. Nat. 133: 157–167. |
| Chrysochloridae | 2 | Stephan, H., Frahm, H. and Baron, G. 1981. New and revisited data on volumes of brain structures in Insectivores and Primates. Folia Primatol. 35: 1–29. |

| | | |
|---|---|---|
| | | McNab, B. K. and Eisenberg, J. F. 1989. Brain size and its relation to the rate of metabolism in mammals. Am. Nat. 133: 157–167. |
| Indriidae | 5 | Bauchot, R. and Stephan, H. 1966. Donnees nouvelles sur l'encephalization des insectivores et des Prosimiens. Mammalia 30: 160–196.<br>Stephan, H., Frahm, H. and Baron, G. 1981. New and revisited data on volumes of brain structures in Insectivores and Primates. Folia Primatol. 35: 1–29.<br>Stephan, H., Baron, G. and Frahm, H. D. 1998. Comparative size of brains and brain components. In: Steklis, H. D. and Erwin, J. (eds), Comparative primate biology, vol. 4: Neurosciences. pp 1–38, Alan R. Liss, Inc., New York.<br>Pérez-Barbería, F. J., Shultz, S. and Dunbar, R. I. M. 2007. Evidence for coevolution of sociality and relative brain size in three orders of mammals. Evolution 61: 2811–2821. |
| Lemuridae | 8 | Hrdlička, A. 1905. Brain weight in vertebrates. Smithsonian Miscellaneous Collections 48:89–112.<br>Hrdlička, A. 1925. Weight of the brain and of the internal organs in american monkeys. Am. J. Phys. Anthropol. 8:201–211.<br>Anthony, R. 1938. Essai de recherche d'une expression anatomique approximative du degré d'organisation cérébrale, autre que le poids de l'encéphale comparé au poids du corps. Bull. Soc. Anthropol. Paris 9:17–67.<br>Crile, G. M. D. and Quiring, D. P. 1940. A record of the body weight and certain organ and gland weights of 3690 animals. Ohio J. Sci. 5: 219–259.<br>Bauchot, R. and Stephan, H. 1966. Donnees nouvelles sur l'encephalization des insectivores et des Prosimiens. Mammalia 30: 160–196.<br>Sacher, G. A. and Staffeldt, E. F. 1974. Relation of gestation time to brain weight for placental mammals: implications for the theory of vertebrate growth. Am. Nat. 108: 593–615.<br>Stephan, H., Frahm, H. and Baron, G. 1981. New and revisited data on volumes of brain structures in Insectivores and Primates. Folia Primatol. 35: 1–29.<br>McNab, B. K. and Eisenberg, J. F. 1989. Brain size and its relation to the rate of metabolism in mammals. Am. Nat. 133: 157–167.<br>Stephan, H., Baron, G. and Frahm, H. D. 1998. Comparative size of brains and brain components. In: Steklis, H. D. and Erwin, J. (eds), Comparative primate biology, vol. 4: Neurosciences. pp 1–38, Alan R. Liss, Inc., New York.<br>Pérez-Barbería, F. J., Shultz, S. and Dunbar, R. I. M. 2007. Evidence for coevolution of sociality and relative brain size in three orders of mammals. Evolution 61: 2811–2821. |
| Lepilemuridae | 2 | Bauchot, R. and Stephan, H. 1966. Donnees nouvelles sur l'encephalization des insectivores et des Prosimiens. Mammalia 30: 160–196.<br>Stephan, H., Baron, G. and Frahm, H. D. 1998. Comparative size of brains and brain components. In: Steklis, H. D. and Erwin, J. (eds), Comparative primate biology, vol. 4: Neurosciences. pp 1–38, Alan R. Liss, Inc., New York.<br>Pérez-Barbería, F. J., Shultz, S. and Dunbar, R. I. M. 2007. Evidence for coevolution of sociality and relative brain size in three orders of mammals. Evolution 61: 2811–2821. |
| Leporidae | 11 | Sacher, G. A. and Staffeldt, E. F. 1974. Relation of gestation time to brain weight for placental mammals: implications for the theory of vertebrate growth. Am. Nat. 108: 593–615.<br>Mace, G. M., Harvey, P. H. and Clutton-Brock T. H. 1981. Brain size and ecology in small mammals. J. Zool. Lond. 193: 333–354. |

| | | |
|---|---|---|
| | | McNab, B. K. and Eisenberg, J. F. 1989. Brain size and its relation to the rate of metabolism in mammals. Am. Nat. 133: 157–167. |
| Lorisidae | 3 | Bauchot, R. and Stephan, H. 1966. Donnees nouvelles sur l'encephalization des insectivores et des Prosimiens. Mammalia 30: 160–196.<br>Sacher, G. A. and Staffeldt, E. F. 1974. Relation of gestation time to brain weight for placental mammals: implications for the theory of vertebrate growth. Am. Nat. 108: 593–615.<br>Stephan, H., Frahm, H. and Baron, G. 1981. New and revisited data on volumes of brain structures in Insectivores and Primates. Folia Primatol. 35: 1–29.<br>McNab, B. K. and Eisenberg, J. F. 1989. Brain size and its relation to the rate of metabolism in mammals. Am. Nat. 133: 157–167.<br>Stephan, H., Baron, G. and Frahm, H. D. 1998. Comparative size of brains and brain components. In: Steklis, H. D. and Erwin, J. (eds), Comparative primate biology, vol. 4: Neurosciences. pp 1–38, Alan R. Liss, Inc., New York.<br>Pérez-Barbería, F. J., Shultz, S. and Dunbar, R. I. M. 2007. Evidence for coevolution of sociality and relative brain size in three orders of mammals. Evolution 61: 2811–2821. |
| Macropodidae | 53 | Sacher, G. A. and Staffeldt, E. F. 1974. Relation of gestation time to brain weight for placental mammals: implications for the theory of vertebrate growth. Am. Nat. 108: 593–615.<br>McNab, B. K. and Eisenberg, J. F. 1989. Brain size and its relation to the rate of metabolism in mammals. Am. Nat. 133: 157–167.<br>Ashwell, K.W.S. 2008. Encephalization of Australian and New Guinean Marsupials. Brain. Behav. Evol. 71:181–199. |
| Macroscelididae | 4 | Sacher, G. A. and Staffeldt, E. F. 1974. Relation of gestation time to brain weight for placental mammals: implications for the theory of vertebrate growth. Am. Nat. 108: 593–615.<br>Stephan, H., Frahm, H. and Baron, G. 1981. New and revisited data on volumes of brain structures in Insectivores and Primates. Folia Primatol. 35: 1–29. |
| Manidae | 1 | McNab, B. K. and Eisenberg, J. F. 1989. Brain size and its relation to the rate of metabolism in mammals. Am. Nat. 133: 157–167. |
| Megachiroptera | 53 | McNab, B. K. and Eisenberg, J. F. 1989. Brain size and its relation to the rate of metabolism in mammals. Am. Nat. 133: 157–167.<br>Baron, G., Stephan, H. and Frahm, H. D. 1996. Comparative neurobiology in Chiroptera. Birkhäuser, Basel. |
| Megalonychidae | 1 | Crile, G. M. D. and Quiring, D. P. 1940. A record of the body weight and certain organ and gland weights of 3690 animals. Ohio J. Sci. 5: 219–259.<br>McNab, B. K. and Eisenberg, J. F. 1989. Brain size and its relation to the rate of metabolism in mammals. Am. Nat. 133: 157–167. |
| Mephitidae | 6 | Gittleman, J. L. 1986. Carnivore brain size, behavioral ecology, and phylogeny. J. Mammal. 67:23–36.<br>Dunbar, R. I. M. and Bever, J. 1998. Neocortex size predicts group size in Carnivores and some Insectivores. Ethology 104: 695–708.<br>Pérez-Barbería, F. J., Shultz, S. and Dunbar, R. I. M. 2007. Evidence for coevolution of sociality and relative brain size in three orders of mammals. Evolution 61: 2811–2821. |

| | | |
|---|---|---|
| Microbiotheriidae | 1 | Ashwell, K.W.S. 2008. Encephalization of Australian and New Guinean Marsupials. Brain. Behav. Evol. 71:181–199. |
| Microchiroptera | 261 | Dubois, E. 1897. Sur le rapport du poids de l'encephale avec le grandeur du corps chez les mammifieres. Bull. Soc. Anthropol. Paris 8: 337–376.<br>Sacher, G. A. and Staffeldt, E. F. 1974. Relation of gestation time to brain weight for placental mammals: implications for the theory of vertebrate growth. Am. Nat. 108: 593–615.<br>McNab, B. K. and Eisenberg, J. F. 1989. Brain size and its relation to the rate of metabolism in mammals. Am. Nat. 133: 157–167.<br>Baron, G., Stephan, H. and Frahm, H. D. 1996. Comparative neurobiology in Chiroptera. Birkhäuser, Basel. |
| Muridae | 67 | Sacher, G. A. and Staffeldt, E. F. 1974. Relation of gestation time to brain weight for placental mammals: implications for the theory of vertebrate growth. Am. Nat. 108: 593–615.<br>Mace, G. M., Harvey, P. H. and Clutton-Brock T. H. 1981. Brain size and ecology in small mammals. J. Zool. Lond. 193: 333–354.<br>McNab, B. K. and Eisenberg, J. F. 1989. Brain size and its relation to the rate of metabolism in mammals. Am. Nat. 133: 157–167. |
| Mustelidae | 34 | Sacher, G. A. and Staffeldt, E. F. 1974. Relation of gestation time to brain weight for placental mammals: implications for the theory of vertebrate growth. Am. Nat. 108: 593–615.<br>McNab, B. K. and Eisenberg, J. F. 1989. Brain size and its relation to the rate of metabolism in mammals. Am. Nat. 133: 157–167.<br>Dunbar, R. I. M. and Bever, J. 1998. Neocortex size predicts group size in Carnivores and some Insectivores. Ethology 104: 695–708.<br>Pérez-Barbería, F. J., Shultz, S. and Dunbar, R. I. M. 2007. Evidence for coevolution of sociality and relative brain size in three orders of mammals. Evolution 61: 2811–2821. |
| Myocastoridae | 1 | Sacher, G. A. and Staffeldt, E. F. 1974. Relation of gestation time to brain weight for placental mammals: implications for the theory of vertebrate growth. Am. Nat. 108: 593–615.<br>Mace, G. M., Harvey, P. H. and Clutton-Brock T. H. 1981. Brain size and ecology in small mammals. J. Zool. Lond. 193: 333–354 |
| Myrmecobiidae | 1 | Ashwell, K.W.S. 2008. Encephalization of Australian and New Guinean Marsupials. Brain. Behav. Evol. 71:181–199. |
| Myrmecophagidae | 3 | Dubois, E. 1897. Sur le rapport du poids de l'encephale avec le grandeur du corps chez les mammifieres. Bull. Soc. Anthropol. Paris 8: 337–376.<br>Anthony, R. 1938. Essai de recherche d'une expression anatomique approximative du degré d'organisation cérébrale, autre que le poids de l'encéphale comparé au poids du corps. Bull. Soc. Anthropol. Paris 9:17–67.<br>Crile, G. M. D. and Quiring, D. P. 1940. A record of the body weight and certain organ and gland weights of 3690 animals. Ohio J. Sci. 5: 219–259.<br>McNab, B. K. and Eisenberg, J. F. 1989. Brain size and its relation to the rate of metabolism in mammals. Am. Nat. 133: 157–167. |
| Mysticeti | 5 | Marino, L. 1998. A Comparison of encephalization between Odontocete Cetaceans and Anthropoid Primates. Brain. Behav. Evol. 51: 230–238. |

| | | |
|---|---|---|
| Nandiniidae | 1 | Gittleman, J. L. 1986. Carnivore brain size, behavioral ecology, and phylogeny. J. Mammal. 67:23–36. |
| Nesomyidae | 4 | Mace, G. M., Harvey, P. H. and Clutton-Brock T. H. 1981. Brain size and ecology in small mammals. J. Zool. Lond. 193: 333–354. |
| Notoryctidae | 1 | Ashwell, K.W.S. 2008. Encephalization of Australian and New Guinean Marsupials. Brain. Behav. Evol. 71:181–199. |
| Octodontidae | 1 | Mace, G. M., Harvey, P. H. and Clutton-Brock T. H. 1981. Brain size and ecology in small mammals. J. Zool. Lond. 193: 333–354.<br>McNab, B. K. and Eisenberg, J. F. 1989. Brain size and its relation to the rate of metabolism in mammals. Am. Nat. 133: 157–167. |
| Odobenidae | 1 | Bininda-Emonds, O. R. P. 2000. Pinniped brain sizes. Mar. Mamm. Sci. 16: 481–488. |
| Odontoceti | 32 | Sacher, G. A. and Staffeldt, E. F. 1974. Relation of gestation time to brain weight for placental mammals: implications for the theory of vertebrate growth. Am. Nat. 108: 593–615.<br>Marino, L. 1998. A Comparison of encephalization between Odontocete Cetaceans and Anthropoid Primates. Brain. Behav. Evol. 51: 230–238.<br>Manger, P. R. 2006. An examination of cetacean brain structure with a novel hypothesis correlating thermogenesis to the evolution of a big brain. Biol. Rev. 81: 293–338.<br>Lefebvre, L., Marino, L., Sol, D., Lemieux-Lefebvre, S. and Arshad, S. 2006. Large brains and lengthened life history periods in Odontocetes. Brain. Behav. Evol. 68: 218–228. |
| Ochotonidae | 3 | Mace, G. M., Harvey, P. H. and Clutton-Brock T. H. 1981. Brain size and ecology in small mammals. J. Zool. Lond. 193: 333–354.<br>McNab, B. K. and Eisenberg, J. F. 1989. Brain size and its relation to the rate of metabolism in mammals. Am. Nat. 133: 157–167. |
| Ornithorhynchidae | 1 | McNab, B. K. and Eisenberg, J. F. 1989. Brain size and its relation to the rate of metabolism in mammals. Am. Nat. 133: 157–167. |
| Otariidae | 13 | Sacher, G. A. and Staffeldt, E. F. 1974. Relation of gestation time to brain weight for placental mammals: implications for the theory of vertebrate growth. Am. Nat. 108: 593–615.<br>Bininda-Emonds, O. R. P. 2000. Pinniped brain sizes. Mar. Mamm. Sci. 16: 481–488. |
| Peramelidae | 11 | McNab, B. K. and Eisenberg, J. F. 1989. Brain size and its relation to the rate of metabolism in mammals. Am. Nat. 133: 157–167.<br>Ashwell, K.W.S. 2008. Encephalization of Australian and New Guinean Marsupials. Brain. Behav. Evol. 71:181–199. |
| Petauridae | 9 | Ashwell, K.W.S. 2008. Encephalization of Australian and New Guinean Marsupials. Brain. Behav. Evol. 71:181–199. |
| Phalangeridae | 17 | McNab, B. K. and Eisenberg, J. F. 1989. Brain size and its relation to the rate of metabolism in mammals. Am. Nat. 133: 157–167.<br>Ashwell, K.W.S. 2008. Encephalization of Australian and New Guinean Marsupials. Brain. Behav. Evol. 71:181–199. |
| Phascolarctidae | 1 | Ashwell, K.W.S. 2008. Encephalization of Australian and New Guinean Marsupials. Brain. Behav. Evol. 71:181–199. |

| | | |
|---|---|---|
| Phocidae | 19 | Sacher, G. A. and Staffeldt, E. F. 1974. Relation of gestation time to brain weight for placental mammals: implications for the theory of vertebrate growth. Am. Nat. 108: 593–615.<br>Bininda-Emonds, O. R. P. 2000. Pinniped brain sizes. Mar. Mamm. Sci. 16: 481–488. |
| Pitheciidae | 4 | Bauchot, R. and Stephan, H. 1969. Encephalisation et niveau evolutif chez les simiens. Mammalia 33: 225–275.<br>Stephan, H., Frahm, H. and Baron, G. 1981. New and revisited data on volumes of brain structures in Insectivores and Primates. Folia Primatol. 35: 1–29.<br>Marino, L. 1998. A Comparison of encephalization between Odontocete Cetaceans and Anthropoid Primates. Brain. Behav. Evol. 51: 230–238.<br>Stephan, H., Baron, G. and Frahm, H. D. 1998. Comparative size of brains and brain components. In: Steklis, H. D. and Erwin, J. (eds), Comparative primate biology, vol. 4: Neurosciences. pp 1–38, Alan R. Liss, Inc., New York.<br>Pérez-Barbería, F. J., Shultz, S. and Dunbar, R. I. M. 2007. Evidence for coevolution of sociality and relative brain size in three orders of mammals. Evolution 61: 2811–2821. |
| Potoroidae | 7 | Ashwell, K.W.S. 2008. Encephalization of Australian and New Guinean Marsupials. Brain. Behav. Evol. 71:181–199. |
| Procaviidae | 3 | Sacher, G. A. and Staffeldt, E. F. 1974. Relation of gestation time to brain weight for placental mammals: implications for the theory of vertebrate growth. Am. Nat. 108: 593–615.<br>McNab, B. K. and Eisenberg, J. F. 1989. Brain size and its relation to the rate of metabolism in mammals. Am. Nat. 133: 157–167.<br>Sigmund, L. 1968. Das Gehirn der Schliefer (Hyracoidea Huxley 1869) als anatomische Grundlagefur den Vergleich mit den Ungulaten (ein Beitrag zur quantitativen Hirnforschung). Acta Societatis Zoologicae Bohemoslovacae 32: 262–271. |
| Procyonidae | 6 | Crile, G. M. D. and Quiring, D. P. 1940. A record of the body weight and certain organ and gland weights of 3690 animals. Ohio J. Sci. 5: 219–259.<br>Sacher, G. A. and Staffeldt, E. F. 1974. Relation of gestation time to brain weight for placental mammals: implications for the theory of vertebrate growth. Am. Nat. 108: 593–615.<br>McNab, B. K. and Eisenberg, J. F. 1989. Brain size and its relation to the rate of metabolism in mammals. Am. Nat. 133: 157–167.<br>Dunbar, R. I. M. and Bever, J. 1998. Neocortex size predicts group size in Carnivores and some Insectivores. Ethology 104: 695–708.<br>Pérez-Barbería, F. J., Shultz, S. and Dunbar, R. I. M. 2007. Evidence for coevolution of sociality and relative brain size in three orders of mammals. Evolution 61: 2811–2821. |
| Pseudocheiridae | 15 | Ashwell, K.W.S. 2008. Encephalization of Australian and New Guinean Marsupials. Brain. Behav. Evol. 71:181–199. |
| Rhinocerotidae | 3 | Pérez-Barbería, F. J., Shultz, S. and Dunbar, R. I. M. 2007. Evidence for coevolution of sociality and relative brain size in three orders of mammals. Evolution 61: 2811–2821. |
| Sciuridae | 67 | Sacher, G. A. and Staffeldt, E. F. 1974. Relation of gestation time to brain weight for placental mammals: implications for the theory of vertebrate growth. Am. Nat. 108: 593–615.<br>Mace, G. M., Harvey, P. H. and Clutton-Brock T. H. 1981. Brain size and ecology in small mammals. J. Zool. Lond. 193: 333–354. |

| | | |
|---|---|---|
| | | McNab, B. K. and Eisenberg, J. F. 1989. Brain size and its relation to the rate of metabolism in mammals. Am. Nat. 133: 157–167. |
| Solenodontidae | 1 | Stephan, H., Frahm, H. and Baron, G. 1981. New and revisited data on volumes of brain structures in Insectivores and Primates. Folia Primatol. 35: 1–29. |
| Soricidae | 30 | Mace, G. M., Harvey, P. H. and Clutton-Brock T. H. 1981. Brain size and ecology in small mammals. J. Zool. Lond. 193: 333–354.<br>Stephan, H., Frahm, H. and Baron, G. 1981. New and revisited data on volumes of brain structures in Insectivores and Primates. Folia Primatol. 35: 1–29.<br>Gittleman, J. L. 1986. Carnivore brain size, behavioral ecology, and phylogeny. J. Mammal. 67:23–36.<br>McNab, B. K. and Eisenberg, J. F. 1989. Brain size and its relation to the rate of metabolism in mammals. Am. Nat. 133: 157–167.<br>Stephan, H., Baron, G. and Frahm, H. D. 1991. Comparative brain research in mammals, volume 1: Insectivora. Springer-Verlag, New York.<br>Monadjem, A. 1998. Relative brain size of some southern African myomorph rodents. S. Afr. J. Zool. 33:47–49. |
| Spalacidae | 2 | Mace, G. M., Harvey, P. H. and Clutton-Brock T. H. 1981. Brain size and ecology in small mammals. J. Zool. Lond. 193: 333–354.<br>McNab, B. K. and Eisenberg, J. F. 1989. Brain size and its relation to the rate of metabolism in mammals. Am. Nat. 133: 157–167. |
| Suidae | 5 | Sacher, G. A. and Staffeldt, E. F. 1974. Relation of gestation time to brain weight for placental mammals: implications for the theory of vertebrate growth. Am. Nat. 108: 593–615.<br>Pérez-Barbería, F. J., Shultz, S. and Dunbar, R. I. M. 2007. Evidence for coevolution of sociality and relative brain size in three orders of mammals. Evolution 61: 2811–2821. |
| Tachyglossidae | 1 | McNab, B. K. and Eisenberg, J. F. 1989. Brain size and its relation to the rate of metabolism in mammals. Am. Nat. 133: 157–167. |
| Talpidae | 9 | Mace, G. M., Harvey, P. H. and Clutton-Brock T. H. 1981. Brain size and ecology in small mammals. J. Zool. Lond. 193: 333–354.<br>Stephan, H., Frahm, H. and Baron, G. 1981. New and revisited data on volumes of brain structures in Insectivores and Primates. Folia Primatol. 35: 1–29.<br>McNab, B. K. and Eisenberg, J. F. 1989. Brain size and its relation to the rate of metabolism in mammals. Am. Nat. 133: 157–167.<br>Stephan, H., Baron, G. and Frahm, H. D. 1991. Comparative brain research in mammals, volume 1: Insectivora. Springer-Verlag, New York. |
| Tapiridae | 2 | Sacher, G. A. and Staffeldt, E. F. 1974. Relation of gestation time to brain weight for placental mammals: implications for the theory of vertebrate growth. Am. Nat. 108: 593–615.<br>Pérez-Barbería, F. J., Shultz, S. and Dunbar, R. I. M. 2007. Evidence for coevolution of sociality and relative brain size in three orders of mammals. Evolution 61: 2811–2821. |

| | | |
|---|---|---|
| Tarsiidae | 2 | Bauchot, R. and Stephan, H. 1966. Donnees nouvelles sur l'encephalization des insectivores et des Prosimiens. Mammalia 30: 160–196.<br>Stephan, H., Frahm, H. and Baron, G. 1981. New and revisited data on volumes of brain structures in Insectivores and Primates. Folia Primatol. 35: 1–29.<br>McNab, B. K. and Eisenberg, J. F. 1989. Brain size and its relation to the rate of metabolism in mammals. Am. Nat. 133: 157–167.<br>Stephan, H., Baron, G. and Frahm, H. D. 1998. Comparative size of brains and brain components. In: Steklis, H. D. and Erwin, J. (eds), Comparative primate biology, vol. 4: Neurosciences. pp 1–38, Alan R. Liss, Inc., New York.<br>Pérez-Barbería, F. J., Shultz, S. and Dunbar, R. I. M. 2007. Evidence for coevolution of sociality and relative brain size in three orders of mammals. Evolution 61: 2811–2821. |
| Tarsipedidae | 1 | Ashwell, K.W.S. 2008. Encephalization of Australian and New Guinean Marsupials. Brain. Behav. Evol. 71:181–199. |
| Tayassuidae | 1 | Pérez-Barbería, F. J., Shultz, S. and Dunbar, R. I. M. 2007. Evidence for coevolution of sociality and relative brain size in three orders of mammals. Evolution 61: 2811–2821. |
| Tenrecidae | 13 | Stephan, H., Frahm, H. and Baron, G. 1981. New and revisited data on volumes of brain structures in Insectivores and Primates. Folia Primatol. 35: 1–29.<br>McNab, B. K. and Eisenberg, J. F. 1989. Brain size and its relation to the rate of metabolism in mammals. Am. Nat. 133: 157–167.<br>Stephan, H., Frahm, H. and Baron, G. 1981. New and revisited data on volumes of brain structures in Insectivores and Primates. Folia Primatol. 35: 1–29. |
| Thryonomyidae | 1 | Mace, G. M., Harvey, P. H. and Clutton-Brock T. H. 1981. Brain size and ecology in small mammals. J. Zool. Lond. 193: 333–354. |
| Thylacomyidae | 1 | Ashwell, K.W.S. 2008. Encephalization of Australian and New Guinean Marsupials. Brain. Behav. Evol. 71:181–199. |
| Tragulidae | 4 | Sigmund, L. 1981. Morphometrische Untersuchungen an Gehirnen der Wiederkauer (Ruminantia, Artiodactyla, Mammalia): 2. Die Hirn-Korpergewichtsbeziehung der Hirschferkel (Tragulidae). Acta universitatis carolinae. Biologica 1979: 447–463.<br>Pérez-Barbería, F. J., Shultz, S. and Dunbar, R. I. M. 2007. Evidence for coevolution of sociality and relative brain size in three orders of mammals. Evolution 61: 2811–2821. |
| Trichechidae | 1 | McNab, B. K. and Eisenberg, J. F. 1989. Brain size and its relation to the rate of metabolism in mammals. Am. Nat. 133: 157–167.<br>O'Shea, T. J. and Reep, R. L. 1990. Encephalization quotients and life-history traits in the Sirenia. J. mammal. 71: 534–543. |
| Tupaiidae | 4 | Dubois, E. 1897. Sur le rapport du poids de l'encephale avec le grandeur du corps chez les mammifieres. Bull. Soc. Anthropol. Paris 8: 337–376.<br>Sacher, G. A. and Staffeldt, E. F. 1974. Relation of gestation time to brain weight for placental mammals: implications for the theory of vertebrate growth. Am. Nat. 108: 593–615.<br>Stephan, H., Frahm, H. and Baron, G. 1981. New and revisited data on volumes of brain structures in Insectivores and Primates. Folia Primatol. 35: 1–29. |

| | | |
|---|---|---|
| | | McNab, B. K. and Eisenberg, J. F. 1989. Brain size and its relation to the rate of metabolism in mammals. Am. Nat. 133: 157–167.<br>Stephan, H., Baron, G. and Frahm, H. D. 1998. Comparative size of brains and brain components. In: Steklis, H. D. and Erwin, J. (eds), Comparative primate biology, vol. 4: Neurosciences. pp 1–38, Alan R. Liss, Inc., New York.<br>Herculano-Houzel, S., Collins, C. E., Wong, P. and Kaas, J. K. 2007. Cellular scaling rules for primate brains. PNAS 104:3562–3567. |
| Ursidae | 8 | Sacher, G. A. and Staffeldt, E. F. 1974. Relation of gestation time to brain weight for placental mammals: implications for the theory of vertebrate growth. Am. Nat. 108: 593–615.<br>Röhrs, Von M., Ebinger, P., Weidemann, W. 1989. Cephalisation bei Viverridae, Hyaenidae, Procyonidae und Ursidae. Z. Zool. Syst. Evol. Forsch. 27:169–180.<br>Dunbar, R. I. M. and Bever, J. 1998. Neocortex size predicts group size in Carnivores and some Insectivores. Ethology 104: 695–708.<br>Pérez-Barbería, F. J., Shultz, S. and Dunbar, R. I. M. 2007. Evidence for coevolution of sociality and relative brain size in three orders of mammals. Evolution 61: 2811–2821. |
| Viverridae | 18 | Gittleman, J. L. 1986. Carnivore brain size, behavioral ecology, and phylogeny. J. Mammal. 67:23–36.<br>McNab, B. K. and Eisenberg, J. F. 1989. Brain size and its relation to the rate of metabolism in mammals. Am. Nat. 133: 157–167.<br>Pérez-Barbería, F. J., Shultz, S. and Dunbar, R. I. M. 2007. Evidence for coevolution of sociality and relative brain size in three orders of mammals. Evolution 61: 2811–2821. |
| Vombatidae | 2 | Ashwell, K.W.S. 2008. Encephalization of Australian and New Guinean Marsupials. Brain. Behav. Evol. 71:181–199. |

Values for the following families were estimated from their sister taxa: Abrocomidae, Anomaluridae, Calomyscidae, Cynocephalidae, Gliridae, Hypsiprymnodontidae, Moschidae, Orycteropodidae, Pedetidae, Petromuridae, Platacanthomyidae, Ptilocercidae.
