# Supplementary material for: Mammalian Collection on Noah's Ark: The Effects of Beauty, Brain and Body Size
Source: PLoS One. 2013 May 15;8(5):e63110. doi: 10.1371/journal.pone.0063110 (PMC3654911; doi:10.1371/journal.pone.0063110)
Supplement: Appendix S4 — Review of mammals reintroduced during the years 1992–2009. We collected journals and book sources of mammalian reintroductions within the years 1992–2009 with detailed information about the reintroduction events. (PDF) [file pone.0063110.s004.pdf]

Appendix 4. Review of mammals reintroduced during the years 1992-2009. We collected journals and book sources of mammalian reintroductions within the years 1992-2009 with detailed information about the reintroduction events.

Reintroduced species	Number of reintroduced species	Number of reintroductions	Attractivenes rank order (out of 123 families)	Relative brain size (EQ) order (out of 123 families)	Reference
**Carnivores:**					Jule, K. R. et al. 2008. The effects of captive expirience on reintroduction survival in carnivores: A review and analysis. Biol. Conserv. 141: 355-363.
**Felidae:**	8	16	2	28	
Lynx canadensis		1			Shenk, T. M. 2001. Post-release monitoring of lynx reintroduced to Colorado. Annual progress report for the U. S. Fish and Wildlife Service. U. S. Department of the Interior, Colorado department of Natural Resources (available at: http://wildlife.state.co.us/Research/Mammal/Lynx/)
Lynx lynx		4			Jule, K. R. et al. 2008. The effects of captive expirience on reintroduction survival in carnivores: A review and analysis. Biol. Conserv. 141: 353–363.<br>Vandel, J. M. et al. 2006. Reintroduction of the lynx into the Vosges mountain massif: from animal survival and movements to population development. Biol. Conserv. 131: 370–385.<br>Červený, J. et al. 1996. Population development and recent distribution of the lynx (Lynx lynx) in the Czech republic. Acta Scientarum Naturalium Academiae Scientiarum Bohemicae Brno 30: 2–15.<br>Breitenmoser, U. et al. 1998. Re-introduction and present status od the lynx (Lynx lynx) in Switzerland. Hysrtix 10: 17–30.
Lynx pardinus		1			Rodriguez, A. 1995. First steps in captive breeding and reintroduction of the Iberian lynx in Spain. Re-Introduction News 11: 14–15.
Lynx rufus		1			Jule, K. R. et al. 2008. The effects of captive expirience on reintroduction survival in carnivores: A review and analysis. Biol. Conserv. 141: 355–363.
Felis concolor		4			Jule, K. R. et al. 2008. The effects of captive expirience on reintroduction survival in carnivores: A review and analysis. Biol. Conserv. 141: 355–363.<br>Ross, P. I. and Jalkotzy, M. G. 1995. Fates of translocated cougars, Felis concolor, in Alberia. The Canadian Field-Naturalist 109: 475–476.<br>Belden, R. C. and Hagedorn, B. W. 1993. Feasibility of translocating panthers into northern Florida. J. Wildl. Manage. 57: 388–397.<br>Jansen, D. and Logan, T. 2002. Improving prospects for the Florida panther. Endangered Spec. Bull. 27: 16–17.
Felis silvestris		1			Jule, K. R. et al. 2008. The effects of captive experience on reintroduction survival in carnivores: A review and analysis. Biol. Conserv. 141: 355–363.
Acinonyx jubatus		2			Jule, K. R. et al. 2008. The effects of captive experience on reintroduction survival in carnivores: A review and analysis. Biol. Conserv. 141: 355–363.<br>Jule, K. R. et al. 2008. The effects of captive experience on reintroduction survival in carnivores: A review and analysis. Biol. Conserv. 141: 355–363.

Panthera tigris		1			Miquelle, D. et al. 2001. Rehabilitation and translocation of two adult female amur Tigers. Wildlife Conservetaion Society & Zoological Society of London. London.
**Canidae:**	3	13	23	15	
Vulpus velox		1			Bremner-Harrison, S. et al. 2004. Behavioural trait assessment as a release criterion: boldness predicts early death in a reintroduction programme of captivebred swift fox (Vulpes velox). Anim. Conserv. 7: 313–320.
Lycaon pictus		8			Woodroffe, R. and Ginsberg, J. R. 1997. The role of captive breeding and reintroduction in wild dog conservation. In: Woodroffe, R. et al. (eds.), The African wild dog: status survey and conservation action plan. IUCN/SSC Canid Specialist Group, Gland, 182 pp.<br>Moehrenschlager, A. and Somers, M. 2004. Canid reintroductions and metapopulation management. In: Sillero-Zubiri, C. et al. (eds.), Canids: foxes, wolves, jackals, and dogs: status survey and conservation action plan. IUCN/SSC Canid Specialist Group, Gland, Switzerland and Cambridge, UK.<br>Graf, J. A. et al. 2006. Evolutionary ecology meets wildlife management: artificial group augmentation in the re-introduction of endangered African wild dogs Lycaon pictus. Anim. Conserv. 9: 398–403.
Canis lupus		4			Phillips, M. K. and Smith, D. W. 1997. Yellowstone wolf project: Biennial report 1995 and 1996 (No. YCR-NR-97-4). National Park Service, Yellowstone Center for Resources, Yellowstone National Park, Wyoming: 28pp.<br>Moehrenschlager, A. and Somers, M. 2004. Canid reintroductions and metapopulation management. In: Sillero-Zubiri, C. et al. (eds.), Canids: foxes, wolves, jackals, and dogs: status survey and conservation action plan. IUCN/SSC Canid Specialist Group, Gland, Switzerland and Cambridge, UK.<br>Jule, K. R. et al. 2008. The effects of captive experience on reintroduction survival in carnivores: A review and analysis. Biol. Conserv. 141: 355–363.
Ursidae:	2	8	4	20	
Ursus amricanus		5			Clark, J. D. et al. 2002. Bear reintroductions: lessons and challenges. Ursus 13: 153–163.<br>Wear, B .J. et al. 2005. Factors affecting settling, survival, and viability of black bears reintroduced to Felsenthal National Wildlife Refuge, Arkansas. Wildl. Soc. Bull. 33: 1363–1374.<br>Eastridge, R. and Clark, J. D. 2001. Black bear reintroductionevalutation of 2 soft-release techniques to reintroduce black bears. Wildl. Soc. Bull. 29: 1163–1174.<br>Stiver, W. H. et al. 1997. Use of pen-reared black bears for augmentation or reintroductions. Int. Conf. Bear. Res. and Manage. 9: 145–150.
Ursus arctos		3			Clark, J. D. et al. 2002. Bear reintroductions: lessons and challenges. Ursus 13: 153–163.
**Mustelidae:**	3	8	33	23	
Lutra lutra		1			Sjoasen, T. 1996. Survivorship of captive-bred and wild-caught reintroduced European otters Lutra lutra in Sweden. Biol. Conserv. 76: 161–165.
Lontra canadensis		2			Jule, K. R. et al. 2008. The effects of captive experience on reintroduction survival in carnivores: A review and analysis. Biol. Conserv. 141: 355–363.<br>Johnson, S. A. and Berkley, K. A. 1999. Restoring river otters to Indiana. Wildl. Soc. Bull. 27: 419–427.
Mustela nigripes		5			Russell, W. C. et al. 1994. The genetic basis of black-footed ferret reintroduction. Conserv. Biol. 8: 263–266.<br>Vargas, A. et al. 1999. Black-footed ferret reproduction and reintroduction in 1998. IUCN – Small Carnivore Conservation Newsletter 20: 32.
**Ungulates:**					

**Bovidae:**	5	8	19	35	
Oryx leucoryx		4			Harding, L. E. et al. 2007. Reintroduction of the Arabian oryx Oryx leucoryx in Jordan: war and redemption. Oryx 41: 478–487.<br>Kiwan, K. et al. 2008: Re-introduction of Arabian oryx into Um Al Zomoul, Abu Dhabi Emirate, United Arab Emirates: 177-180. In: Global re-introduction perspectives – Re-introduction case-studies from around the globe. Soorae, P. S. (ed.), IUCN/SSC Re-introduction Specialist Group (RSG). c/o Environment Agency-Abu Dhabi. United Arab Emirates 2008: 284 pp.<br>Saltz, D. 2008. Re-introduction of Arabian oryx into the Negev Desert, Israel: 185-187 In: Global re-introduction perspectives – Re-introduction case-studies from around the globe. Soorae, P. S. (ed.), IUCN/SSC Re-introduction Specialist Group (RSG). c/o Environment Agency-Abu Dhabi. United Arab Emirates 2008: 284 pp.<br>Simkis, G. 2008. Re-introduction of Arabian Oryx into the Dubai Desert Conservation Reserve, Dubai, UAE: 188-191 In: Global re-introduction perspectives – Re-introduction case-studies from around the globe. Soorae, P. S. (ed.), IUCN/SSC Re-introduction Specialist Group (RSG). c/o Environment Agency-Abu Dhabi. United Arab Emirates 2008: 284 pp.
Ourebia ourebi		1			Grey-Ross, R. et al. 2009. Reintroduction failure of captive-bred oribi (Ourebia ourebi). S. Afr. J. Wildl. Res. 39: 34–38.
Gazella gazella		1			Dunham, K. M. 1996.Population growth of mountain gazelles Gazella gazella reintroduced to Central Arabia. Biol. Conserv. 81: 205–214.
Bison bonasus		1			Belousova, I. P. et al. 2005. Reintroduction of the European Bison into the Forest Ecosystem of the Orlovskoe Poles'e National Park. Russian. J. Ecol. 36: 115–119.
Bison bison		1			Larte, N. C. et al. 1999. Dynamics of reintroduction in an indigenous large ungulate: the wood bison of northern Canada. Anim. Conserv. 4: 299–309.
**Cervidae:**	2	3	10	19	
Cervus elaphus corsicanus		1			Kidjo, N. et al. 2007. Extirpation and reintroduction of the Corsican red deer Cervus elaphus corsicanus in Corsica. Oryx 41: 488–494.
Dama mesopotamica		2			Zidon, R. et al. 2008. Behavioral changes, stress, and survival following reintroduction of Persian fallow deer from two breeding facillities. Conserv. Biol. 23: 1026–1035.<br>Bar-David, S. et al. 2005. Demographic models and reality in reintroductins: Persian fallow deer in Israel. Conserv. Biol. 19: 131–138.
**Equidae:**	1	3	7	24	
Equus caballus przewalskii		3			Boyd, L. and Bandi, N. 2002. Reintroduction of takhi, Equus ferus przewalskii, to Hustai National Park, Mongolia: time budget and synchrony of activity pre- and post-release. Appl. Anim. Behav. Sci. 78: 87–102.<br>Kůs, E. 2004. Výroční zpráva 2004 - Chovatelství - kůň Převalského. ZOO Praha (avaliable at: http://www.zoopraha.cz/cs/o-zoo/vyrocni-zpravy/vyrocni-zprava-2004/chovatelstvi-kun-prevalskeho), accessed 1.5.2010<br>Chen, J. et al. 2007. Reproduction and development of the released Przewalski's horses (Equus przewalskii) in Xinjiang, China. J. Equine. Sci. 19: 1–7.<br>Kůs, E. 2009. Národní park Gobi B. Ochrana přírody 2: 30–32.<br>Souris, A. C. et al. 2006. Time budget-, behavioral synchrony- and body score development of a newly released Przewalski's horse group Equus ferus przewalskii, in the Great Gobi B strictly protected area in SW Mongolia. Appl. Anim. Behav. Sci. 107: 307–321.
**Rodents:**					
**Muridae:**	1	1	68	47	

Arvicola terrestris		1			Mathews, F. et al. 2006. Health surveillance in wildlife reintroductions. Biol. Conserv. 131: 338–347.
**Castoridae:**	1	2	31	93	
Castor fiber		2			Vorel, A. and Kostkan, V. 2005. Rešerše a hodnocení relizovaných a probíhajících projektů aktivní ochrany bobra evropského (Castor fiber) v České republice: 407–413. In: Kumstátová, T. et al. (eds), Hodnocení projektů aktivní podpory ohrožených živočichů v České republice. Praha: 432 pp.
**Sciuridae:**	2	4	12	42	
Spermophilus citellus		3			Hulová, Š. 2005. Rešerše a hodnocení relizovaných a probíhajících projektů aktivní ochrany sysla obecného (Spermophilus citellus) v České republice: 397–405. In: Kumstátová, T. et al. (eds), Hodnocení projektů aktivní podpory ohrožených živočichů v České republice. Praha: 432 pp.
Sciurius vulgaris		1			Shuttleworth, C. et al. 2008. Re-introduction of the red squirrel into Newborough forest on the island of Anglesey, UK: 163–166 – In: Global re-introduction perspectives – Re-introduction case-studies from around the globe. Soorae, P. S. (ed.), IUCN/SSC Re-introduction Specialist Group (RSG). c/o Environment Agency-Abu Dhabi. United Arab Emirates 2008: 284 pp.
Primates:					
**Hominidae:**	2	3	50	4	
Pan troglodytes		1			Huffman, M. A. et al. 2008. Introduction of chimpanzees onto Rubondo Island National Park, Tanzania: 213–216 – In: Global re-introduction perspectives – Re-introduction case-studies from around the globe. Soorae, P. S. (ed.), IUCN/SSC Re-introduction Specialist Group (RSG). c/o Environment Agency-Abu Dhabi. United Arab Emirates 2008: 284 pp.
Gorilla gorilla		2			King, T. and Courage, A. 2008. Western gorilla re-introduction to the Batéké Plateau region of Congo and Gabon: 217–220 – In: Global re-introduction perspectives – Re-introduction case-studies from around the globe. Soorae, P. S. (ed.), IUCN/SSC Re-introduction Specialist Group (RSG). c/o Environment Agency-Abu Dhabi. United Arab Emirates 2008: 284 pp.
**Lemuridae:**	1	1	26	17	
Lemur catta		1			Keith-Lucas, T. et al. 1999. Changes in behavior in free-ranging Lemur catta following release in a natural habitat. Am. J. Primatol. 47: 15–28.
